# ASIC3-dependent metabolomics profiling of serum and urine in a mouse model of fibromyalgia

**DOI:** 10.1038/s41598-019-48315-w

**Published:** 2019-08-20

**Authors:** Wei-Hsiang Hsu, Cheng-Han Lee, Yen-Ming Chao, Ching-Hua Kuo, Wei-Chi Ku, Chih-Cheng Chen, Yun-Lian Lin

**Affiliations:** 1 Department of Chinese Pharmaceutical Sciences and Chinese Medicine Resources, China Medical University, Taichung, 40402 Taiwan; 2 Institute of Biomedical Sciences, Academia Sinica, Taipei, 115 Taiwan; 3 Department of Pharmacy, National Taiwan University, Taipei, 100 Taiwan; 4 School of Medicine, College of Medicine, Fu Jen Catholic University, New Taipei, 24205 Taiwan; 5 Taiwan Mouse Clinic – National Comprehensive Mouse Phenotyping and Drug Testing Center, Academia Sinica, Taipei, 115 Taiwan

**Keywords:** Chronic pain, Predictive markers, Neuropathic pain

## Abstract

Fibromyalgia (FM) is characterized by chronic widespread pain. The pathogenesis of FM remains unclear. No specific biomarkers are available. Animal models of FM may provide an opportunity to explore potential biomarkers in a relative homogenous disease condition. Here, we probed the metabolomics profiles of serum and urine in a mouse model of FM induced by intermittent cold stress (ICS). We focused on the role of acid-sensing ion channel 3 (ASIC3) in the metabolomics profiling because ICS treatment induced chronic widespread muscle pain lasting for 1 month in wild-type (*Asic3*^+/+^) but not *Asic3*-knockout (*Asic3*^−/−^) mice. Serum and urine samples were collected from both genotypes at different ICS stages, including before ICS (basal level) and post-ICS at days 10 (middle phase, P10) and 40 (recovery phase, P40). Control naïve mice and ICS-induced FM mice differed in ^1^H-NMR- and LC-MS-based metabolomics profiling. On pathway analysis, the leading regulated pathways in *Asic3*^+/+^ mice were taurine and hypotaurine, cysteine and methionine, glycerophospholipid, and ascorbate and aldarate metabolisms, and the major pathways in *Asic3*^−/−^ mice involved amino acid-related metabolism. Finally, we developed an algorithm for the impactful metabolites in the FM model including *cis*-aconitate, kynurenate, taurine, pyroglutamic acid, pyrrolidonecarboxylic acid, and 4-methoxyphenylacetic acid in urine as well as carnitine, deoxycholic acid, lysoPC(16:0), lysoPC(20:3), oleoyl-*L*-carnitine, and trimethylamine *N*-oxide in serum. *Asic3*^−/−^ mice were impaired in only muscle allodynia development but not other pain symptoms in the ICS model, so the ASIC3-dependent metabolomics changes could be useful for developing diagnostic biomarkers specific to chronic widespread muscle pain, the core symptom of FM. Further pharmacological validations are needed to validate these metabolomics changes as potential biomarkers for FM diagnosis and/or treatment responses.

## Introduction

Fibromyalgia (FM) is characterized by chronic widespread musculoskeletal pain affecting 2% to 8% of the adult population, with high prevalence in women^1^. In 2010, the American College of Rheumatology proposed a revised version of the fibromyalgia impact questionnaire (FIQR) for FM diagnosis. The FIQR assesses the presence of pain at 19 sites on a body diagram (widespread pain index, WPI) and measures the symptom severity (SS) with a score (0–3) for 3 core symptoms (insomnia, fatigue and cognitive impairment) and an average score (0–3) for additional somatic symptoms, which are summed to create an SS overall score. However, a precise diagnosis of FM is still challenging. To effectively diagnose FM and evaluate the therapeutic efficacy, reliable biomarkers for FM are indispensible but not yet available.

Animal models of FM with good face and predictive validity could provide an opportunity to explore potential metabolic biomarkers in a relative homogenous disease^2,3^. Intermittent cold stress (ICS) is one of well-established mouse model of FM, in which widespread and long-lasting muscle allodynia is induced after a sudden and frequent change of environment temperature. The predictive validity of the ICS model mimics the clinical observation of FM because the ICS-induced pain can be resolved by administration of antidepressants (e.g., milnacipran) or anticonvulsants (e.g., gabapentinoids) but poorly responds to morphine^4–6^. Similar to FM comorbidities, ICS-treated mice also show fatigue, anxiety-like behaviors, and cognitive impairments^7,8^. In addition, ICS-induced mechanical allodynia was attenuated in gonadectomized male but not gonadectomized female mice, which may be correlated with the predominance of fibromyalgia in females^4^. Thus, ICS is an ideal animal model of FM to investigate the underlying mechanisms of chronic widespread pain as well as possible diagnostic biomarkers and a therapeutic strategy.

Recently, metabolites quantification has great biological interest for finding possible biomarkers of neurological diseases because the identity and abundance of metabolites directly reflect the outcome of the genome, transcriptome and proteome, which may be perturbed by neurological disorders^9,10^. Metabolic profiling can involve use of high-resolution nuclear magnetic resonance (NMR) and mass spectrometry (MS). With the maturity of powerful techniques, metabolomics is a useful approach for searching for biomarkers and revealing the underlying mechanisms of FM.

Acid-sensing ion channel 3 (ASIC3) is an important pain transducer predominantly expressed in muscle nociceptors and involved in pain-associated tissue acidosis, such as inflammatory pain, rheumatoid arthritis, post-operative pain, chest pain, postoperative pain, and so on^11–13^. Previous studies of rodent models induced by repeated intramuscular acid injection have shown that ASIC3 is involved in FM-like pain^14–16^. Therefore, lacking ASIC3 signaling would impair chronic muscle pain development in the ICS model and change the metabolomics profiling in serum and urine. We profiled the metabolomics of the *Asic3* wild-type and -knockout mouse models at different stages of ICS treatment to identify ASIC3-dependent metabolic changes. We aimed to probe meaningful metabolic biomarkers that could be used for diagnosis of FM.

## Results

### *Asic3* knockout specifically impaired ICS-induced chronic muscle pain in mice

**Figure 1:**
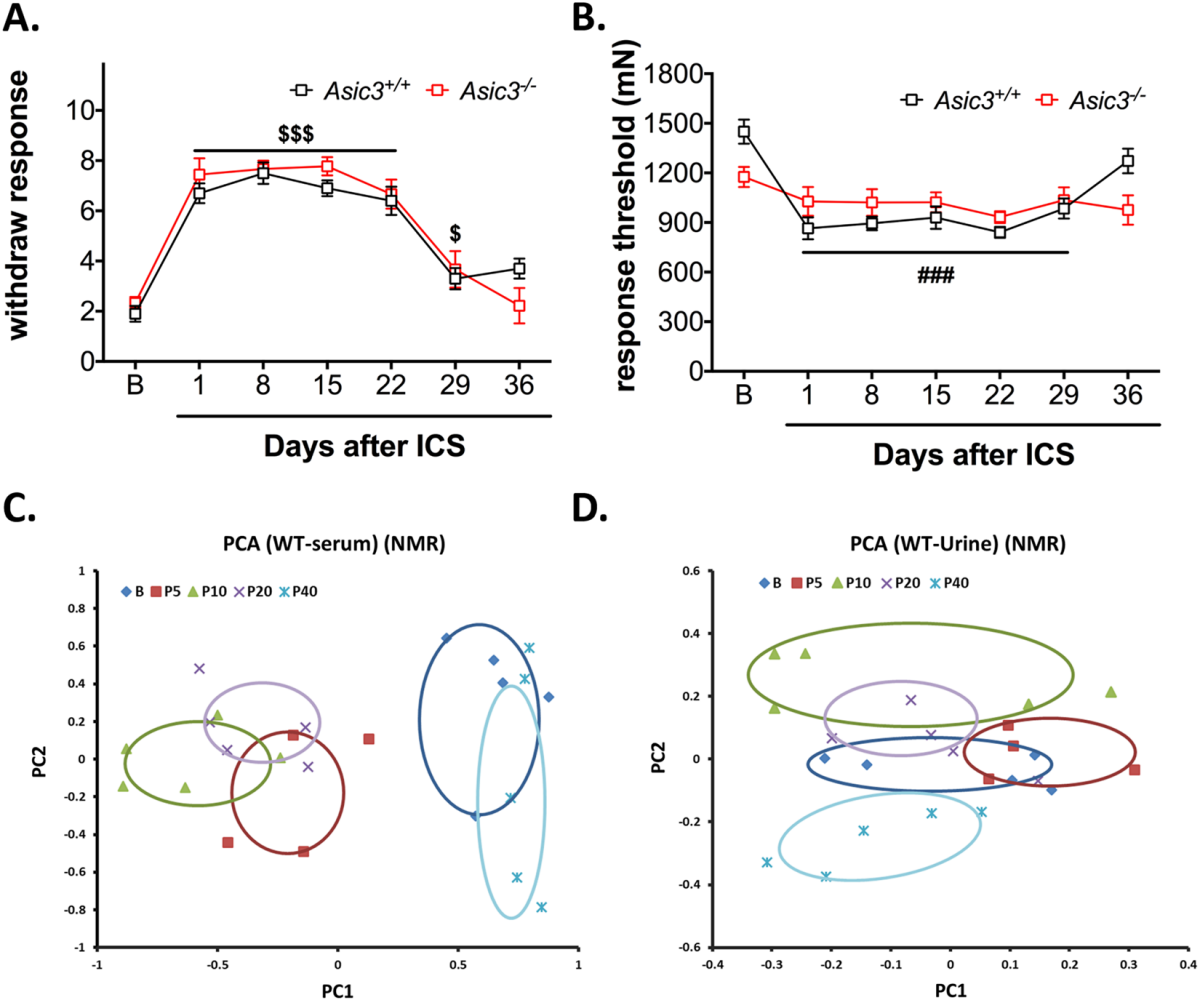
Effects of intermittent cold stress (ICS) on mechanical sensitivity and metabolites of mice. (**A**) ICS-induced paw mechanical hyperalgesia. Data were analyzed by 2-way ANOVA [interaction F_(6,102)_ = 1.477, P = 0.194; time F_(6,102)_ = 53.18, P < 0.001; genotype F_(1,17)_ = 0.384, P = 0.544], followed by post-hoc Bonferroni multiple comparison test. ^$^*p* < 0.05, ^$$$^*p* < 0.001, compared to basal group. (**B**) ICS-induced muscle mechanical allodynia. Data were analyzed by 2-way ANOVA [interaction F(6,102) = 4.446, P < 0.001; time F(6,102) = 9.878, P < 0.001; genotype F(1,17) = 0.025, P = 0.877], followed by post-hoc Bonferroni multiple comparison test. ^###^*p* < 0.001 compared to basal level in *Asic3*^+/+^ mice. (**C**,**D**) Principal component analysis (PCA) of serum and urine metabolites of *Asic3*^+/+^ mice. ^1^H-NMR–based metabolites were identified by metabolomic comparison among mice at pre-ICS (B, n = 5), and post-ICS days: P5 (n = 4), P10 (n = 5), P20 (n = 5), and P40 (n = 5). PCA plots were based on ^1^H-NMR data for serum (**C**) and urine (**D**).

Although ASIC3 is involved in the development of chronic widespread muscle pain in an FM model induced by repeated intramuscular acid injection, its role in other FM models is unknown. To validate whether ASIC3 is involved in the development of stress-induced systemic musculoskeletal pain, we compared ICS-induced mechanical hyperalgesia of hind paws and muscle allodynia between *Asic3*^+/+^ and *Asic3*^−/−^ mice. As expected, ICS induced long-lasting paw mechanical hyperalgesia and muscle mechanical allodynia in *Asic3*^+/+^ mice up to 29 days (Fig. 1A,B). In contrast, *Asic3*^−/−^ mice showed ICS-induced paw hyperalgesia similar to *Asic3*^+/+^ mice but not muscle allodynia (Fig. 1A,B). Of note, naïve *Asic3*^+/+^ and *Asic3*^−/−^ mice did not differ in basal sensitivity to mechanical stimuli on paw and muscle from ages 7 to 8 weeks and at 40 days after ICS, although mechanical withdrawal threshold of muscle was lower for *Asic3*^−/−^ than *Asic3*^+/+^ mice (Supplementary Fig. 1A,B). These results suggest a role for ASIC3 in the stress-induced chronic widespread muscle pain.

### NMR- and LC-MS-based metabolomics for evaluating the metabolic profile after ICS treatment

Because ICS-induced muscle pain can last for about 29 days, we can monitor the metabolic changes of mice in different pain states: from pre-ICS (B) to post-ICS treatment at the early phase (P5), middle phase (P10), late phase (P20), and recovery phase (P40). Metabolomics is an effective post-genomic research tool that can have applications in many disciplines, including the study of disease progression. Thus, we obtained global untargeted serum and urine profiles in mice after ICS by ^1^H-NMR spectroscopy. The spectral data were submitted to multivariate statistical analysis to identify changes of circulating metabolites during the different phases of the experiment. Serum and urine metabolomics analyses were performed with 24 *Asic3*^+/+^ mice with or without ICS treatment (n = 5, 4, 5, 5, and 5 for pre-ICS, P5, P10, P20, and P40, respectively). Principal component analysis (PCA) was used to obtain an overview of the data and to identify intrinsic clustering and possible outliers in the data. In our pilot study, the metabolite profiling during pain progression was similar to that from biological function testing (Fig. 1A,B) and the profile between pre-ICS (B) and P10 showed a remarkable difference in both serum and urine (Fig. 1C,D). Of note, we used another set of naïve mice as untreated controls and found no change in metabolic profiling between mice at ages 7 to 8 weeks (age-matched with pre-ICS, n = 5) and 13 to 14 weeks (age-matched with P40, n = 5) (data not show). Therefore, we chose B and P10 for the following ^1^H-NMR and LC-MS metabolomic approaches. As well, analysis of metabolites by LC-MS is highly complementary to metabolite profiling by ^1^H-NMR. To find more metabolites, UPLC-QTOF-MS metabolomic study was further performed. We randomly selected three pre-ICS (B) mouse groups and three P10 groups for LC-MS analysis.

**Figure 2:**
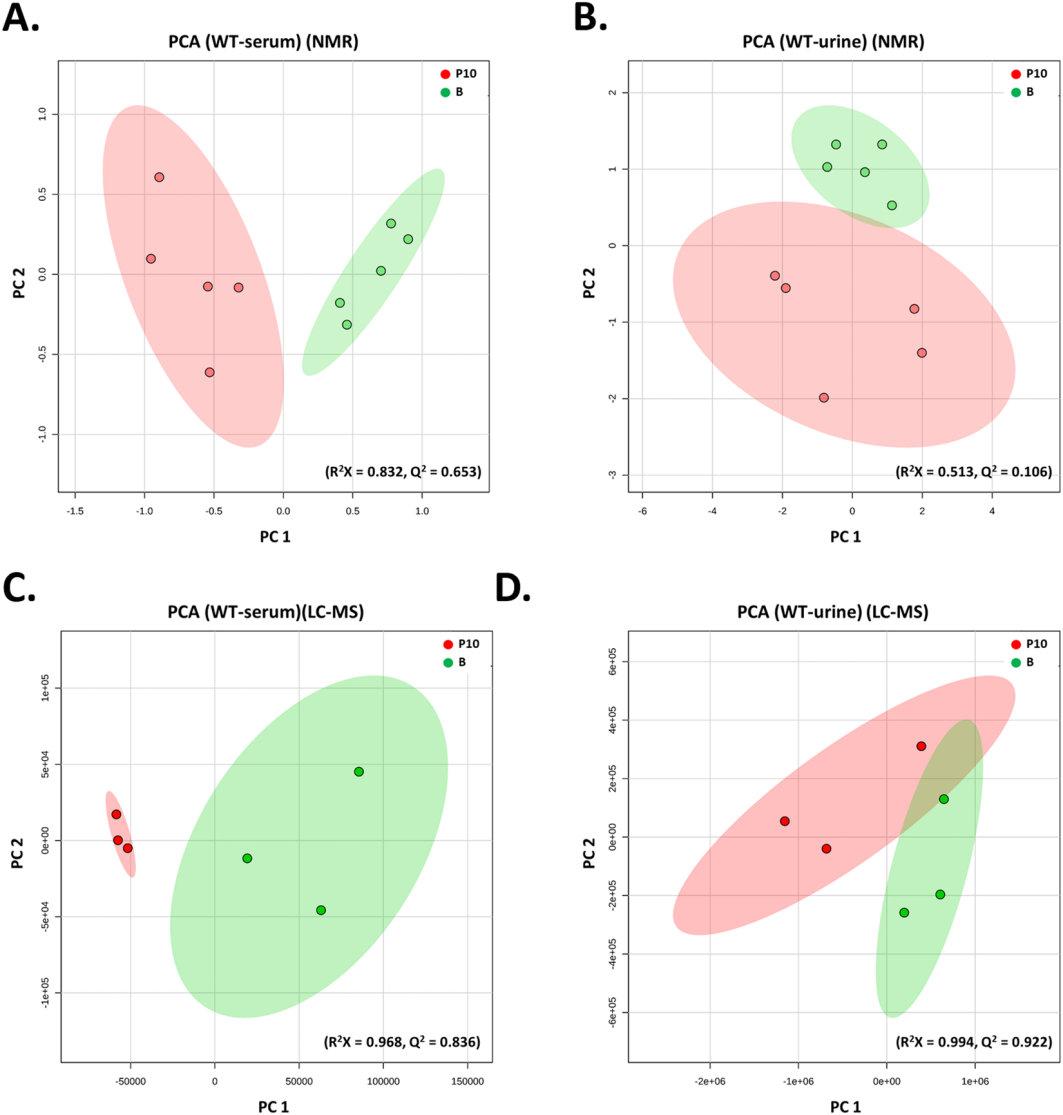
PCA analysis of metabolites in serum and urine from *Asic3*^+/+^ mice [ICS-induced experimental fibromyalgia model (P10) vs controls (B)]. ^1^H-NMR- and LC-MS/MS-based metabolites were identified by metabolomic comparison between P10 and B. PCA plots were based on ^1^H-NMR data for serum (**A**) and urine (**B**) from B (green) and P10 groups (red) and LC-MS/MS data for serum (**C**) and urine (**D**).

PCA was used to analyze molecular features identified from B and P10 samples by 1H-NMR and LC-MS (Fig. 2). The plot of principal component 1 (PC1) versus PC2 showed a separation between B and P10. To obtain a more reliable statistical analysis and specific loadings, a PLS-DA model was used to discriminate samples from B and P10 groups. A reasonably good separation was obtained in the scatter plot (Supplementary Fig. 2). These plots from serum and urine data showed a significant separation in metabolomes between the control and ICS-treated groups in *Asic3*^+/+^ mice.

### Metabolic profiles and untargeted metabolites in serum and urine from ICS-treated *Asic3*^+/+^ mice

With combined ^1^H-NMR and LC-MS results, about 150 and 200 metabolites were detected in serum and urine, respectively, of ICS-treated *Asic3*^+/+^ mice; 34 and 47 metabolites showed significantly changed expression in serum and urine after ICS-induction (Supplementary Tables 1 and 2). Furthermore, changed levels of 4-methoxyphenylacetic acid, creatine, galacturonic acid, glucuronate, glycerol 3-phosphate, *L*-3-phenyllactic acid, and uridine were detected in both serum and urine. Of them, 4-methoxyphenylacetic acid and *L*-3-phenyllactic acid were upregulated in both serum and urine. The expression of creatine, galacturonic acid, glucuronate, glycerol 3-phosphate, and uridine in serum was opposite to that in urine. Moreover, abnormal expression of some metabolites were recovered at P40 in ICS-treated *Asic3*^+/+^ mice (Supplementary Tables 1 and 2).

### Metabolic profiles and untargeted metabolites of serum and urine in ICS-treated *Asic3*^−/−^ mice

**Figure 3:**
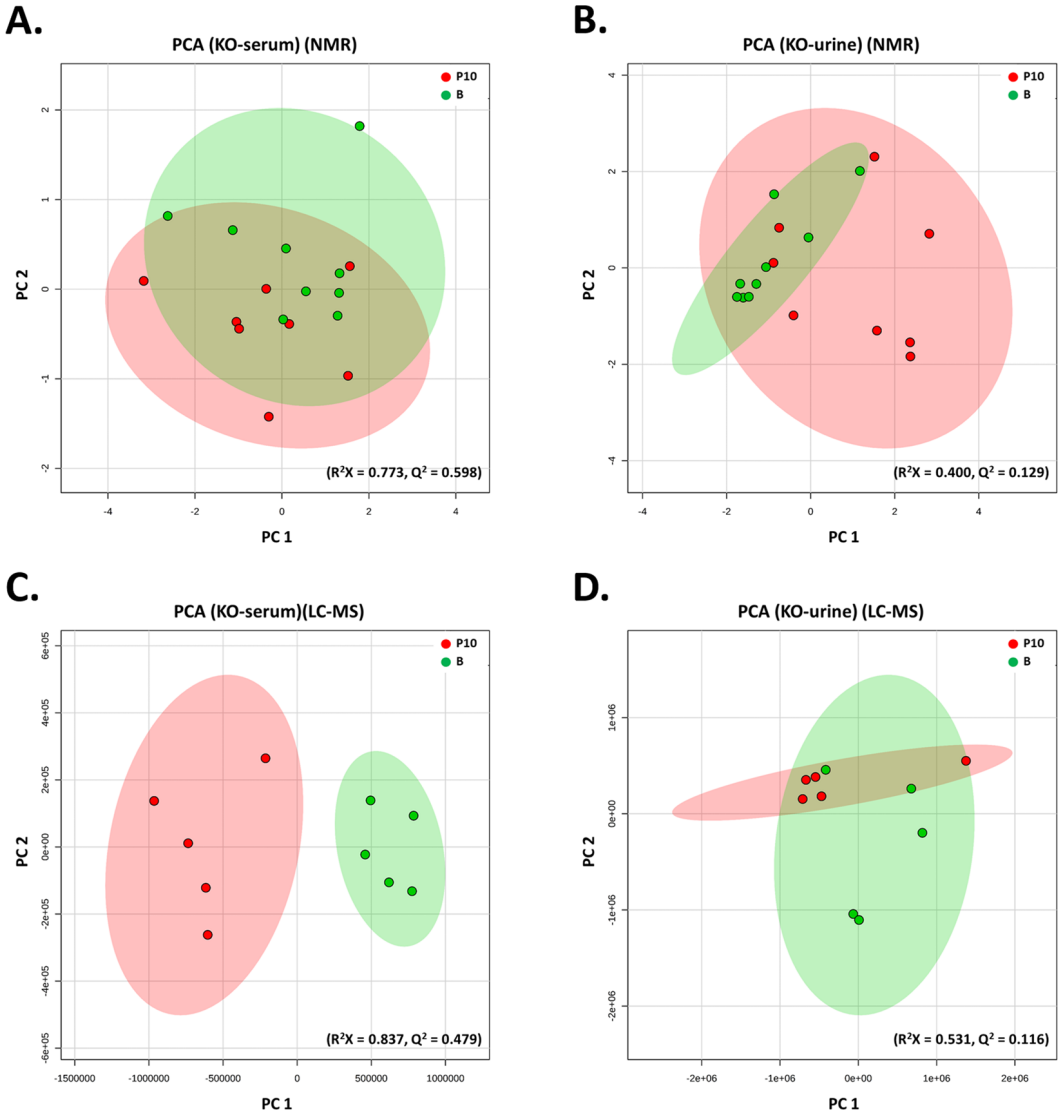
PCA analysis of *Asic3*^−/−^ mice [ICS-induced experimental fibromyalgia model (P10) vs controls (B)]. ^1^H-NMR– and LC-MS/MS–based metabolites were identified by metabolomic comparison between P10 and B. PCA plots were based on ^1^H-NMR data for serum (**A**) and urine (**B**) from B (green) and P10 groups (red) and LC-MS/MS data for serum (**C**) and urine (**D**).

Since *Asic3*^−/−^ mice showed no ICS-induced muscle pain but retained mechanical hyperalgesia in cutaneous tissue, we wondered how ICS would affect the metabolic profiling in *Asic3*^*−/−*^ mice. Again, multivariate analysis of metabolomics separated B and P10 metabolites in both serum and urine of *Asic3*^−/−^ mice. PCA and PLS-DA analysis of ^1^H-NMR and LC-MS data showed a trend of inter-group separation on the plots in Fig. 3 and Supplementary Fig. 3.

With combined ^1^H-NMR and LC-MS, the expression of 70 and 52 metabolites significantly changed in serum and urine, respectively, of ICS-induced *Asic3*^−/−^ mice (Supplementary Tables 3 and 4). Of them, 2-oxoglutarate, 5-methylcytidine, hydroxyphenyllactic acid, methylmalonate, valine, and betaine was increased in both serum and urine. However, the levels of butyrylcarnitine, carnitine, decenoyl-L-carnitine, dopamine, hexanoylcarnitine, isobutyrylcarnitine, rhamnose, taurine, and thymidine in serum was opposite to that in urine.

### Comparison of metabolomics between *Asic3*^+/+^ and *Asic3*^−/−^ mice after ICS induction

**Figure 4:**
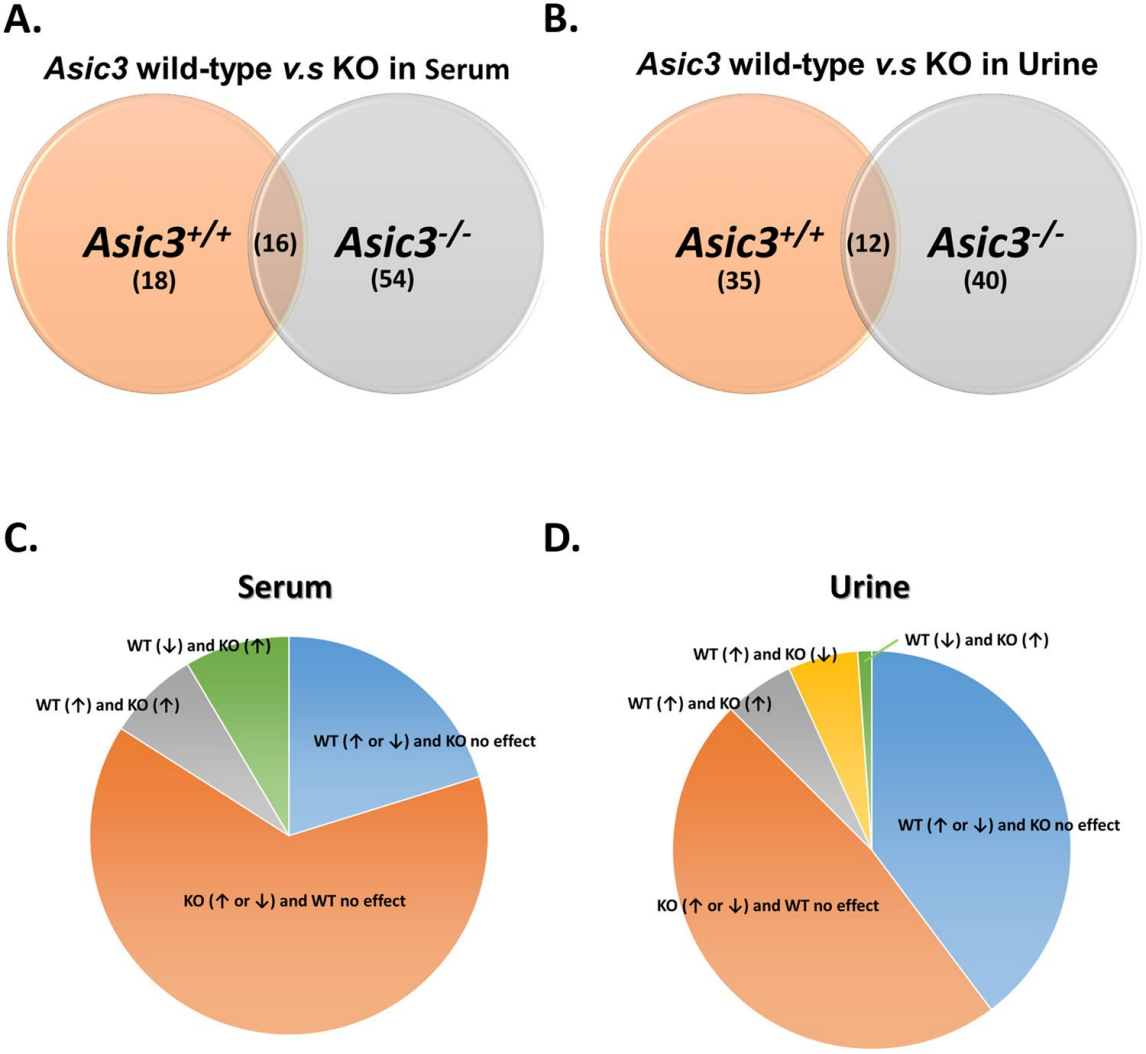
Change in ICS-induced metabolites between *Asic3*^+/+^ and *Asic3*^−/−^ mice. (**A**,**B**) Venn diagram of change in serum and urine. (**C**,**D**) Compartmentalization of change in serum and urine.

We next probed the ASIC3-dependent metabolic profiling in the ICS model by comparing the metabolomics results for *Asic3*^+/+^ and *Asic3*^−/−^ mice. The effect of *Asic3*^−/−^ on ICS-induced metabolomics might provide useful insights to identify specific biomarkers for chronic muscle pain development. On multivariate analysis, with NMR or LC-MS, both R^2^ and Q^2^ values were higher in *Asic3*^+/+^ than *Asic3*^−/−^ mice (serum: 0.832 vs 0.773 and 0.653 vs 0.598, respectively, for NMR, 0.968 vs 0.837 and 0.836 vs 0.479 for LC-MS; urine: 0.513 vs 0.400 and 0.106 vs 0.129 for NMR, 0.994 vs 0.531 and 0.922 vs 0.116 for LC-MS). These results showed that the metabolomics were more sensitive to ICS treatment for *Asic3*^+/+^ than *Asic3*^−/−^ mice. We could discriminate B and P10 phases after ICS treatment, 16 metabolites were found in both *Asic3*^+/+^ and *Asic3*^−/−^ mouse serum (intersection area) (Fig. 4A,C); the levels of alanine, carnitine, creatine, decenoyl-L-carnitine, linoleyl-L-carnitine, lysoPC(20:2), lysoPC(20:3), propionyl-L-carnitine in *Asic3*^+/+^ mice were opposite to those in *Asic3*^−/−^ mice. The levels of 4-methoxyphenylacetic acid, butyrylcarnitine, hydroxyphenyllactic acid, iso-butyrylcarnitine, methylmalonate, L-3-phenyllactic acid, succinate, and lysoPC (18:3) were increased in both *Asic3*^+/+^ and *Asic3*^−/−^ mouse serum after ICS treatment (Fig. 4A).

In urine, 12 metabolites, including 3-pyridylacetic acid, 5-methylcytidine, D-ribose 5-phosphate, lactic acid, glutaric acid, citric acid, dopamine, glucuronate, taurine, thymidine, methylmalonate, and phenylacetylglycine, were commonly changed in both *Asic3*^+/+^ and *Asic3*^−/−^ mice (Fig. 4B,D). The levels of dopamine, glucuronate, taurine, thymidine, and methylmalonate in *Asic3*^+/+^ mice was contrary to that of *Asic3*^−/−^ mice. 3-pyridylacetic acid, 5-methylcytidine, D-ribose 5-phosphate, glutaric acid, lactic acid, and citric acid were higher at the P10 phase, but phenylacetylglycine was lower at the P10 phase in both mouse types (Fig. 4B).

### Metabolomic-related pathways and networks are affected by ICS treatment

**Figure 5:**
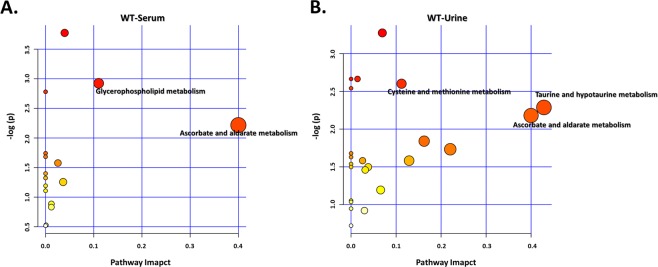
Pathway and network analysis of differentially expressed metabolites in ICS-treated *Asic3*^+/+^ mice. Network pathway was identified by MetPA software. Metabolism was inferred from changes in levels of intermediates during substance metabolism in serum (**A**) and urine (**B**) of mice.

**Figure 6:**
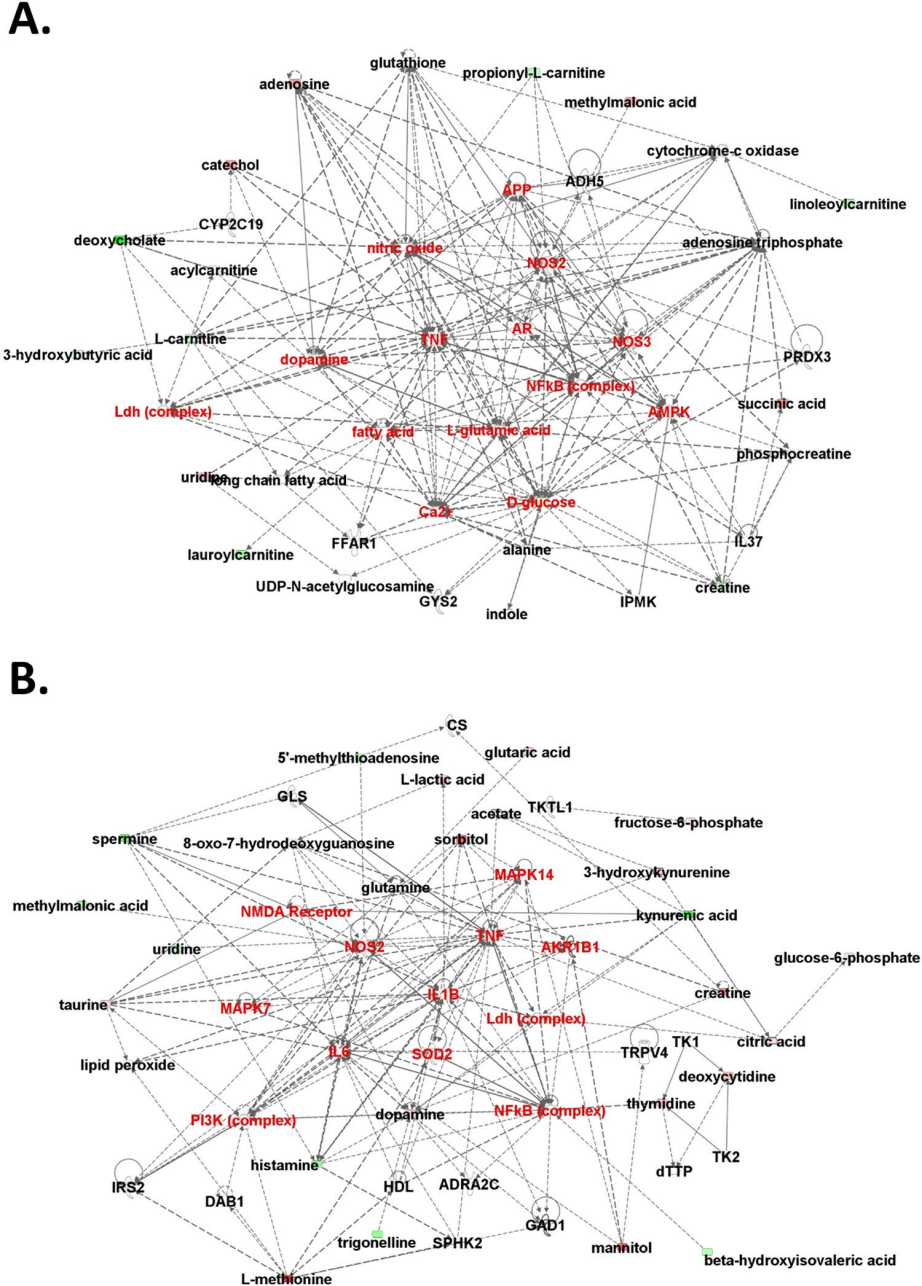
Network analysis of differentially expressed metabolites in ICS-treated *Asic3*^+/+^ mice. Molecular network involved using ingenuity pathway tools in serum (**A**) and urine (**B**). Direct interactions are represented by continuous lines and indirect interactions by dashed lines. The red nodes represent upregulated metabolites and green nodes downregulated metabolites. The red words are hot spots of network.

To reveal the most relevant pathways affected by ICS treatment, we used metabolic pathway analysis (MetPA) with MetaboAnalyst 4.0, which involves pathway analysis through pathway enrichment and pathway topological analyses, to further explore the impact of these metabolites with changed expression and to identify possible biochemical pathways that are affected in FM-like conditions. To identify the most relevant metabolic pathways, the impact-value threshold calculated from pathway topology analysis was set to 0.1. The impact value and −log(p) with MetPA were used to evaluate the importance of the pathways in the development of ICS-induced chronic widespread pain (Figs 5 and 6). As a whole, the leading pathways that work with ICS treatment were taurine and hypotaurine, cysteine and methionine, glycerophospholipid, and ascorbate and aldarate metabolisms. The common pathway shared in serum and urine involved ascorbate and aldarate metabolism (Fig. 5).

Next, IPA was applied to identify the biochemical pathways responsible for the observed metabolic abnormalities. In the network analysis, ICS treatment-related metabolites in serum and urine tended to gather in a single network (Fig. 6). Accordingly, within these two metabolomics networks, dopamine, Ldh complex, NOS2, TNF, and NF-κB complex were common hot spots. These results agree with a previous study showing FM patients with strong expression of NF-κB and NF-κB-dependent pro-inflammatory cytokine generation^17,18^. The other hot spots in the two networks were also highly correlated.

We demonstrated that metabolites with altered expression in ICS-induced *Asic3*^−/−^ mice were responsible for the dominant influencing pathways, including tyrosine metabolism and biosynthesis of branched chain amino acids (BCAAs: valine, leucine and isoleucine) and aromatic amino acids (phenylalanine, tyrosine and tryptophan) **(**Supplementary Fig. 4A,B). These pathways are all amino acid-related metabolomic pathways. In *Asic3*^−/−^ mice, levels of valine, leucine and isoleucine were increased (Supplementary Tables 3 and 4), hence BCAA biosynthesis was upregulated. A previous study showed that BCAAs could increase the antinociceptive effect^19^. This finding could parallel in part the restricted ICS-induced abnormal pain in *Asic3*^−/−^ mice.

### Potential ASIC3-dependent FM biomarkers from the ICS mouse model

**Figure 7:**
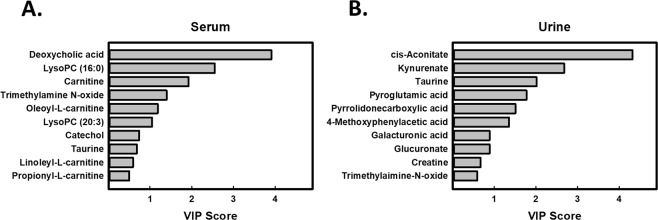
Variable importance in projection (VIP) scores from PLS-DA model of serum and urine from ICS-induced *Asic3*^+/+^ mice. VIP scores analysis based on the weighted coefficients of the PLS-DA model were used to rank the contribution of metabolites to discriminating between ICS treatment (P10) and controls (**B**) by LC-MS/MS in serum (**A**) and urine (**B**).

Supplementary Tables 1–4 provide a comprehensive list of metabolites with significantly changed expression in serum and urine of ICS-treated mice. To generate a list of serum metabolites with changed expression (list A), identified as ASIC3-dependent, ICS-affected metabolites, we could exclude metabolites that were concordant between *Asic3*^+/+^ and *Asic3*^−/−^ mice: 4-methoxyphenylacetic acid, butyrylcarnitine, hydroxyphenyllactic acid, iso-butyrylcarnitine, methylmalonate, *L*-3-phenyllactic acid, succinate, and lysoPC (18:3) (Supplementary Table 1). Then to create a new profile of urine metabolites with changed expression (list B), identified as ASIC3-dependent, ICS-affected metabolites, we could eliminate other metabolites that were concordant between *Asic3*^+/+^ and *Asic3*^−/−^ mice: 3-pyridylacetic acid, 5-methylcytidine, D-ribose 5-phosphate, glutaric acid, lactic acid, citric acid and phenylacetylglycine (Supplementary Table 2). Next, to determine which metabolites mainly contributed to the discrimination between ICS-induced FM and controls, we used variable importance in projection (VIP) analysis for lists A and B. Metabolites with high VIP contribute highly to class separation, whereas those with low VIP provide contribute less. Metabolites with VIP cut-off ≥1 could discriminate the potential biomarkers of FM. We selected 6 metabolites for each model that fulfilled both VIP >1 and P < 0.05 in serum and urine. Serum biomarker candidates were carnitine, deoxycholic acid, lysoPC (16:0), lysoPC (20:3), oleoyl-L-carnitine, and trimethylamine *N*-oxide (TMAO), and urine biomarker candidates were *cis*-aconitate, kynurenate, taurine, pyroglutamic acid, pyrrolidonecarboxylic acid, and 4-methoxyphenylacetic acid (Fig. 7).

## Discussion

FM syndrome represents a complex cluster of symptoms of unknown etiology that include chronic fatigue and pain processing within central and peripheral nervous systems as well as endocrine and muscular systems. In the ICS model, animals are under cold stress accompanied by a high frequency of temperature changes to trigger long-lasting widespread pain conditions, including mechanical allodynia on muscle and hind paws as well as intense thermal hyperalgesia, which mimic the clinical symptoms of FM^4^. In this study, we demonstrated that ASIC3 is required for the ICS-induced chronic muscle pain and identified ASIC3-dependent metabolomics changes in serum and urine of ICS-treated mice as potential biomarkers for FM. *Asic3*^−/−^ mice were impaired in only muscle allodynia development but not other pain symptoms in the ICS model, so the ASIC3-dependent metabolomics changes could be useful for developing diagnostic biomarkers specific to chronic widespread muscle pain, the core symptom of FM^20^.

ASIC3 is so far the most sensitive ion channel responsible for extracellular acidification, with pH_50_ 6.5–7.1, and its acid sensitivity can be potentiated by lactic acid increase that occurs during the hypoxic/ischemic condition^21–23^. Likewise, ASIC3 can be activated by lysoPCs (EC_50_ = 4.3 μM) and potentiated by many pro-inflammatory mediators such as 5HT (EC_50_ = 41 μM), agmatine (EC_50_ = 5 mM), arachidonic acid (EC_50_ = ~5 μM), and RFamide (EC_50_ = 10–50 μM)^24^. Accumulating evidence has shown that ASIC3 is an important pain transducer involved in pain associated with tissue acidosis and exercise-induced muscle fatigue^11,25^. ASIC3 is predominantly expressed in the somatosensory system, including nociceptors and proprioceptors, and macrophages^25–28^. ICS treatment leads to abnormalities in skeletal muscle, with decreased number of capillary contacts per muscle fiber and an increase in damaged mitochondria^29^. These changes indicate that ICS would reduce the distribution of oxygen, nutritive materials, and hormones to muscle fibers and thus impair muscle energy metabolism, as shown in FM^30^. Thus, ASIC3 of muscle nociceptors and resident macrophages in muscle is most likely required for the development of chronic muscle pain and thus affects the metabolomics of serum and urine. Because ASIC3 is predominantly expressed in muscle afferents as compared with skin afferents^31,32^, ICS treatment increases the metabolomics production of lysoPCs that stimulate ASIC3 channels and leads to muscle allodynia. In contrast, an ASIC3-independent pathway would contribute to the ICS-induced paw hyperalgesia and associated metabolomics changes.

Carnitine and its acyl esters (acylcarnitines) play a critical role in energy balance across cell membranes and energy metabolism of tissues that derive much of their energy from fatty acid oxidation^33,34^. Carnitines have indirect and direct oxygen radical scavenging activity and can protect muscle mitochondria against free radical-induced damage^35,36^. Recently, oxidative stress with lipid peroxidation induced by reactive oxygen species has been proposed as a relevant event in the pathogenesis of FM^37,38^. Previous studies have shown low levels of carnitine and acylcarnitines in the blood or muscles causing chronic fatigue, a core symptom of FM^39^. Consistent with the concept that FM pain could result from muscle energy deficiency^40^, lower levels of carnitine and propionyl-L-carnitine in the serum of ICS-treated *Asic3*^+/+^ mice would impair lipid metabolism and compel cells to look for alternate pathways for energy production in the event of an energy crisis^41^. Hence, we found increased levels of some energy producers, such as glycerol 3-phosphate, methylmalonate, and succinate, in ICS-treated *Asic3*^+/+^ mice. Nonetheless, levels of carnitine and propionyl-*L*-carnitine were increased and level of glycerol 3-phosphate was reduced in serum of ICS-treated *Asic3*^−/−^ mice. In addition, ICS-treated *Asic3*^+/+^ mice showed differential expression of acylcarnitines, such as propionyl-*L*-carnitine, decenoyl-*L*-carnitine, linoleyl-*L*-carnitine, oleoyl-*L*-carnitine, and tetradecanoyl-*L*-carnitine. This finding could indicate a reservoir of acyl moieties for the reacylation of membrane phospholipids, a process catalyzed by glycerophospholipid acyltransferase, and thus regulate the levels of lysophosphatidic acid, lysoPC, phosphatidic acid, phosphatidylinositol, and phosphatidylcholine, involved in glycerophospholipid metabolism^42^.

Accumulating evidence has revealed that the lysoPC-platelet-activating factor (PAF)/PAF receptor (PAFr) system plays a role in modulating pain signaling^43,44^. In FM, metabolomics analysis and modeling has suggested a lysoPC-PAFr interaction^45^. LysoPCs containing myristic and palmitic acid may result from the action of PAF-acetylhydrolase on oxidation products of phosphocholine after oxidative stress^46^. LysoPCs, as proinflammatory phospholipids, can generate superoxide anions in neutrophils^47^. Also, lysoPCs can activate the transcription factor NF-κB to regulate immune and inflammatory pathways and induce interleukin 8 (IL-8) and cyclooxygenase-2-dependent interleukin 6 (IL-6) expression and secretion^48,49^. Previous studies demonstrated higher serum IL-6 and IL-8 levels in FM patients than healthy controls^50,51^. Of note, arachidonic acid (AA), which is an important precursor for prostaglandins and lysoPCs, could potentiate ASIC1 and ASIC3 activation and be one of major pro-algesic factors in the sensation of acid-induced pain^52^. Another study also showed that lysoPCs and AA are endogenous activators of ASIC3 in the absence of any extracellular acidification^53^. Marra *et al*. reported that some lysoPCs, such as lysoPC (16:0) or lysoPC (18:1), selectively activate ASIC3 channels of nociceptors and evoke pain in rodents^53^. Pierluigi Caboni *et al*. reported elevated content of lysoPC (14:0), lysoPC (16:0), and lysoPC (18:0) in FM patients^45^, and we found increased serum content of lysoPC (14:0), lysoPC (16:0), and lysoPC (18:3) in *Asic3*^+/+^ mice with ICS treatment. Thus, ICS treatment could increase the metabolomics production of some lysoPCs to stimulate ASIC3 channels, leading to pain hypersensitivity. Of note, our data reveals that with ICS treatment, the serum content of lysoPC (20:2) and lysoPC (20:3) was decreased in *Asic3*^+/+^ mice but increased in *Asic3*^−/−^ mice.

Another major metabolomics finding in the ICS model was changed amino acid and glucose metabolism. The urinary levels of taurine, creatine, lactic acid, and TMAO were higher in ICS-induced *Asic3*^+/+^ mice as compared with naïve mice. However, ICS treatment did not affect the urinary levels of creatine and lactic acid but lowered those of taurine and TMAO in *Asic3*^−/−^ mice. These results agree well with a recent study showing significant increases in urinary levels of metabolites, including hippuric acid, succinic acid, lactic acid, taurine, creatine, and TMAO, in 18 FM patients (vs 20 healthy controls)^54^. As well, an algorithm consisting of succinic acid, taruine, and creatine has been developed for FM diagnosis, with good accuracy^54^. ICS treatment increased the urinary content of pyroglutamic acid in our *Asic3*^+/+^ but not *Asic3*^−/−^ mice. The increased urinary level of pyroglutamic acid might be concurrent with reduced glutathione level and indicate increased oxidative stress^55^. Oxidative stress could be a risk factor in the pathophysiology of FM^38^. Moreover, we detected higher urinary level of dopamine in ICS-treated *Asic3*^+/+^ mice as compared with naïve mice, but no significant change in urinary level of dopamine in ICS-treated *Asic3*^−/−^ mice, which suggests a differential disturbance of dopaminergic transmission in the nervous system between genotypes. In FM patients, the release of endogenous dopamine was disrupted in response to tonic experimental pain in the basal ganglia, so FM patients might show an abnormal release of dopamine in response to non-painful stimulation^56^. Additionally, ICS-treated *Asic3*^+/+^ mice showed reduced urinary level of kynurenate, one of the products of tryptophan metabolism^57^, but the urinary level of kynurenate was not affected in our ICS-treated *Asic3*^−/−^ mice. Of note, low urinary level of tryptophan is associated with depression in humans^58,59^, a common comorbidity among FM patients^60^.

Serum levels of some metabolites, including 4-methoxyphenylacetic acid, butyrylcarnitine, hydroxyphenyllactic acid, iso-butyrylcarnitine, methylmalonate, *L*-3-phenyllactic acid, succinate, and lysoPC (18:3), were increased in both *Asic3*^+/+^ and *Asic3*^−/−^ mice after ICS treatment. Urinary levels of 3-pyridylacetic acid, 5-methylcytidine, D-ribose 5-phosphate, glutaric acid, lactic acid, citric acid, and phenylacetylglycine were increased in both *Asic3*^+/+^ and *Asic3*^−/−^ mice after ICS treatment. As compared with metabolites associated with ASIC3-dependent muscle pain, these consentaneous metabolites are ASIC3-independent and may be associated with ICS-induced mechanical hyperalgesia of hind paws in both *Asic3*^+/+^ and *Asic3*^−/−^ mice.

Pathway analysis exhibited that the affected pathway in ICS-treated *Asic3*^+/+^ mice was taurine and hypotaurine, cysteine and methionine, glycerophospholipid, and ascorbate and aldarate metabolisms. Glycerophospholipid as well as ascorbate and aldarate metabolisms were found associated with hyperalgesia^61^; taurine and hypotaurine metabolisms were related to seizures^62^. Patients with seizure can show high urinary levels of taurine^63^. However, although psychogenic nonepileptic seizure is one of the comorbidities of FM, this association is largely unknown and requires further investigation^64,65^. We found a significant increase in urinary content of pyrrolidonecarboxylic and pyroglutamic acids after ICS treatment. These acids are cyclic derivatives of glutamic acid, which could facilitate the release of acetylcholine and γ-amino butyric acid (GABA) from the cerebral cortex to other biofluids and thus modulate pain signaling^66^. Patients with seizure can show high levels of GABA in serum and urine^67^. Our results may provide new insights to associate FM with seizures.

Understanding the ICS-changed metabolomics in ASIC3-dependent and -independent pathways would provide useful information to further classify the highly heterogeneous FM patients based on metabolomics phenotyping. In this study, we used metabolic profiling of ICS-induced FM mouse models to describe a global metabolic characteristic of FM-like pain symptoms by ^1^H-NMR and LC-MS approaches. The related pathophysiological processes reflected by the metabolic profile of the FM mouse model mainly include taurine and hypotaurine, cysteine and methionine, glycerophospholipid, and ascorbate and aldarate metabolisms. The methodology revealed several potential biomarkers, some not previously described, such as *cis*-aconitate, kynurenate, taurine, pyroglutamic acid, pyrrolidonecarboxylic acid, and 4-methoxyphenylacetic acid in urine as well as carnitine, deoxycholic acid, lysoPC (16:0), lysoPC (20:3), oleoyl-*L*-carnitine, and TMAO in serum.

Nevertheless, the current study has several limitations in the model used and lack of pharmacological validation. Although ICS is an animal model mimicking the symptoms of fibromyalgia, we should never consider that an animal model of fibromyalgia can reflect all aspects of fibromyalgia. Fibromyalgia is a multifactorial pathology with a lack of specific etiologically identified processes^60^. In the ICS model, the induction of chronic widespread pain is somewhat similar to patients who work in a storeroom^68–70^. Because the continuous physical stress can neither be avoided nor controlled, storeroom workers could develop chronic widespread pain due to abnormal levels of serotonin, noradrenalin and dopamine in the central nervous system and an imbalanced autonomic nervous system similar to the pathogenesis of fibromyalgia^71–74^. Thus, we focused on ICS-induced “pain symptoms” in this study and tried to link the metabolomics changes with the ASIC3-dependent and ASIC3-independent pathway. Of note, the metabolomics change could be ASIC3-dependent but independent of pain. Further pharmacological validations are needed to validate these metabolomics changes as potential biomarkers for FM diagnosis and/or treatment responses.

## Materials and Methods

### Mice

8- to 14-week-old female C57BL6/J mice were used in all experiments and 4~5 mice were housed in a group at temperature-controlled environment with 12 hr light/dark cycle from 8:00 to 20:00. Asic3 knockout (*Asic3*^−/−^) mice were generated in our lab as previously described^26^. Wild-type (*Asic3*^+/+^) and *Asic3*^−/−^ mice used in this study were offspring from *Asic3*^*+/−*^ mice bred in a congenic C57BL6/J background. A total of 86 mice were used for the entire experiments; 10 mice were repeatedly used in both behavioral tests and metabolomics analysis (B40 group) (Table 1). For behavioral measurement, 10 *Asic3*^+/+^ mice and 9 *Asic3*^*−/−*^ mice were used for testing mechanical responses in the ICS group, and 6 *Asic3*^+/+^ mice and 5 *Asic3*^−/−^ mice were tested without ICS. For NMR analysis, we used 34 *Asic3*^+/+^ mice, which were separated as B (basal; pre-ICS), n = 10; B40 (39–40 days after basal), n = 5; P5 (ICS-P5), n = 4; P10 (ICS-P10), n = 5; P20 (ICS-P20), n = 5; P40 (ICS-P40), n = 5. For NMR analysis, we also used 32 *Asic3*^−/−^ mice, which were separated as B, n = 14; B40, n = 5; P10, n = 8; and P40, n = 5. Then, for untargeted metabolite screening (LC-MS analysis), we selected from 34 *Asic3*^+/+^ mice (the same as for NMR analysis) separated as B, n = 3; B40, n = 5; P10, n = 3; and P40, n = 3, as well as 32 *Asic3*^−/−^ mice (the same as for NMR analysis) separated as B, n = 5; B40, n = 5; P10, n = 5; and P40, n = 5.

**Table 1:** Number of mice used in behavior and metabolomics studies.

Genotype	*Asic3* ^+/+^								*Asic3* ^−/−^					
Behavior	B and B40		ICS					Total	B and B40		ICS			Total
	6*		10					16	5*		9			14
Metabolomics	B	B40	B (pre-ICS)	P5	P10	P20	P40	Total	B	B40	B (pre-ICS)	P10	P40	total
NMR	5	5*	5	4	5	5	5	34	5	5*	5	9	8	32
LC-MS	5	5*	3		3		3		5	5*	5	5	5	

*The mice used for B40 NMR and LC-MS were the same as those used for B and B40 behavior.

All procedures followed the Guide for the Care and Use of Laboratory Animals published by the US National Institutes of Health (NIH publication No. 85–12, revised 1996) and were approved by the Institutional Animal Care and Utilization Committee of Academia Sinica.

### Intermittent cold stress (ICS)

Female mice were used in the ICS treatment because sex hormones would affect the development of mechanical allodynia in male mice^4^. Two 8-week-old female C57BL/6 J mice (18~20 g) were habituated on a stainless-steel mesh platform in a plexiglass cage (L × W × H = 15 × 12.5 × 14.5 cm) for 4 days. A 2-day ICS treatment was adopted^4^. In brief, mice were kept in the 4 ± 2 °C (cold room) overnight from 16:30 to 10:00 the next day, then stayed alternately in rooms with 24 ± 2 °C (room temperature) and 4 ± 2 °C every 30 min for 6 hr as a run of ICS treatment. During the ICS treatment, food pellets and 1% agar were placed on the mesh floor of the cage. After 2 days of ICS treatment, mice were housed in individually ventilated cages at room temperature. Serum and urine were collected at different ICS stages, including before ICS (basal level, B) and post-ICS at days 5 (P5), 10 (P10), 20 (P20), and 40 (P40). Mechanical responses of hind paws and gastrocnemius muscle (GM) were measured before ICS (B) and post-ICS at days 1, 8, 15, 22, 29, and 36.

### Urine and serum collection

Spot urine samples were collected before serum collection and immediately stored at −80 °C until use. For serum preparation, blood was collected from the submandibular vessel of mice punctured by a lancet and stored in a 1.5-ml microcentrifuge tube. To collect serum as much as possible for NMR and LC-MS analysis, serum was collected from each mouse at only one time. The collected blood samples were kept at room temperature for clotting for 15 min, then centrifuged twice at 1200 *g* for 10 min at 4 °C to completely remove clotted debris. All serum samples were stored at −80 °C until use.

### Measurement of paw mechanical responses

The paw withdrawal response was measured by the von Frey test. Briefly, after mice habituated on a stainless-steel mesh, 0.02 g von Frey fiber was applied five times on each mouse hind paw at 1-min intervals or longer. Positive responses were defined as the target hind limb showing toe spread or withdrawal, flinching, shaking and licking behavior. The sum of positive withdrawal responses from both hind paws indicated the mechanical sensitivity of the mouse.

### Measurement of muscle mechanical responses

The muscle withdraw threshold was measured by using the Pressure Application Measurement (PAM) system (Ugo Basile, VA, Italy) with a modified cone-shape, rounded probe (2.23 mm diameter). Mice were trained to adapt to the stress of the restraint and pressure application once a day for at least 2 days. The probe was slowly compressed on the belly of the gastrocnemius muscle (GM) until hind limb withdrawal and then released immediately. The maximum compression force to induce hind-paw withdrawal was recorded and defined as the mechanical threshold of the GM. Two trials for each hind limb were performed at 5-min intervals. For each mouse, the mechanical withdrawal threshold of the GM was estimated from the average of all trials tested with both hind limbs. The mechanical sensitivity of the mouse in all time points was normalized to the mechanical threshold at the basal level.

### Metabolome profile and untargeted metabolites extraction by ^1^H-NMR and LC-MS

^1^H-NMR analysis was performed mainly as described^75^. For LC-MS analysis, a 50-μl serum sample was extracted with 200 μl methanol by shaking at 1000 rpm for 2 min on a Geno/Grinder 2010 (SPEX SamplePrep, Metuchen, NJ, USA). The mixture was then centrifuged at 15000 *g* for 5 min at 4 °C. The supernatant was evaporated until dryness. The residue was re-reconstituted in 50% methanol and sonicated for 10 min, then centrifuged at 15000 *g* for 5 min at 4°C. The supernatant was used for LC-MS/MS analysis. For urine samples, using 200 μl water and 250 μl methanol instead of 200 μl methanol. The LC-MS analyses was performed by an Agilent 1290 UPLC system (ACQUITY UPLC HSS T3 column, 2.1 × 100 mm; 1.8 µm; Waters, Milford, MA, USA) coupled with the 6540-Quadrupole-Time-of-Flight (QTOF) mass system (Agilent Technologies, Santa Clara, CA, USA). The LC mobile phase consisted of 0.1% formic acid in water (solvent A) and acetonitrile (ACN; solvent B). The gradient profile was run at 0–1.5 min: 2% B; 1.5–9 min: linear gradient from 2 to 50% B; 9–14 min: linear gradient from 50 to 95% B; 14–15 min: 95% B. The flow rate was 0.3 ml min^−1^. The injection volume was 2 μL. A jet stream electrospray ionization source was used for sample ionization. The following parameters were used throughout the study: curtain gas: gas temperature (325 °C), gas flow (8 L/min), nebulizer (40 psi), sheath gas temperature (325 °C), sheath gas flow (10 L/min), capillary voltage (40 kV for positive and 35 kV for negative), and fragmenter (120 V). The mass scan range was m/z = 50–1700.

### Lysophosphatidylcholine targeted metabolite in serum by LC-MS

The sample preparation was similar to the above method but using 100 µL deionized water and 1000 µL methanol/chloroform (1/2) for 100 µL serum as the extracting solvent^76^. The lower organic layer was collected and dried with nitrogen gas and reconstituted in 120 µL of 100% methanol for LC-MS analysis.

LC-MS was run on a ZORBAX Eclipse Plus C18, RRHD, 2.1 × 100 mm, 1.8 µm column (Agilent Technologies, Waldbronn, Germany) for QTOF and a mobile phase consisted of A, 10 mM ammonium acetate in 0.1% aqueous formic acid and B, 0.1% formic acid with 10 mM ammonium acetate in ACN/isopropyl alcohol (50/50) with a 0.35-mL min^−1^ linear gradient elution: 0–2.0 min, 35% to 80% mobile phase B; 2.0 to 7 min, 80% to 100% mobile phase B; 7 to 14 min, 100% mobile phase B; and column re-equilibration with 100% mobile phase B for 2 min. The sample reservoir and column oven were maintained at 4 °C and 55 °C, respectively. The injection volume was 5 μl. The positive electrospray ionization mode involved 300 °C dry gas temperature, 5 L min^−1^ dry gas flow rate, 45 psi nebulizer pressure, 250 °C sheath gas temperature, 11 L min^−1^ sheath gas flow rate, 3500 V capillary voltage, and 500 V nozzle voltage. MS acquisition was executed in precursor ion scan (PIS) mode.

All UPLC-MS raw data were converted to mzXML format by using Trapper (ISB) and normalized by TIPick, an in-house package, as well as peak enhancement and peak chosen for the targeted metabolites^77,78^. An in-house database of lysophosphatidylcholine in the Metabolomics Core Laboratory, Center of Genomic Medicine, National Taiwan University, was used for screening.

### Metabolic pathway and network analysis in metaboanalyst and IPA

MetaboAnalyst 4.0 (http://www.metaboanalyst.ca) is a suite of metabolomics tools that includes modules for multivariate statistical analysis, metabolite enrichment analysis (MSEA), and metabolite pathway analysis (MetPA). MetPA was used to sort the metabolites with significantly altered expression on ICS treatment into biologically relevant metabolic pathways.

Ingenuity Pathways Analysis (IPA; http://www.ingenuity.com) is a web-based software used to identify the biological pathways and functions relevant to bio-molecules of interest. To scrutinize the systematic influence of treatment related metabolites, metabolites interaction networks were generated from the knowledge sorted in the Ingenuity Pathway Knowledge Base from the uploaded metabolite lists and the changed direction of these metabolites^79^.

### Statistical analysis

Statistical analysis of behavior measurements involved two-way repeated-measures ANOVA followed by a post-hoc Bonferroni test. Multivariate statistical analysis including principal component analysis (PCA) and partial least-squares discriminant analysis (PLS-DA) was used to analyze data from MS or NMR spectra and the response variable by SIMCA-P^+^ v12.0 (Umetrics, Umeå, Sweden). All analyses involved using IBM SPSS v23.0. Descriptive statistics are presented as mean ± SD, median (range), or number (%). Student’s *t*, Fisher’s exact, or chi-square test was used to compare groups. Paired *t* test or Wilcoxon signed-rank test was used to compare paired data. All calculated *p*-values were two-tailed. Statistical significance was defined at *p* < 0.05.

## Supplementary information

Supplementary Figures and Tables
