# Supplementary material for: ASIC3-dependent metabolomics profiling of serum and urine in a mouse model of fibromyalgia
Source: Sci Rep. 2019 Aug 20;9:12123. doi: 10.1038/s41598-019-48315-w (PMC6702159; doi:10.1038/s41598-019-48315-w)
Supplement: Supplementary file 1 — Supplementary Figures and Tables [file 41598_2019_48315_MOESM1_ESM.docx]

***ASIC3-dependent metabolomics profiling of serum and urine in a mouse model of fibromyalgia***

Wei-Hsiang Hsu^1,#^, Cheng-Han Lee^2,#^, Yen-Ming Chao^1^, [Ching-Hua Kuo](http://rx.mc.ntu.edu.tw/myDOP/SCENE/FACULTY/facultyview.php?malangue=EN&rub=faculty//1//c4ca4238a0b923820dcc509a6f75849b8946432fasXvktje1lFk)^3^, Wei-Chi Ku^4^, Chih-Cheng Chen,^2,5,#^, Yun-Lian Lin^1,^*

^1^ Department of Chinese Pharmaceutical Sciences and Chinese Medicine Resources, China Medical University, Taichung 40402, Taiwan

^2^ Institute of Biomedical Sciences, Academia Sinica, Taipei 115, Taiwan

*^3^* Department of Pharmacy, National Taiwan University, Taipei 100, Taiwan

*^4^* School of Medicine, College of Medicine, Fu Jen Catholic University, New Taipei 24205, Taiwan

^5^Taiwan Mouse Clinic – National Comprehensive Mouse Phenotyping and Drug Testing Center, Academia Sinica, Taipei 115, Taiwan

^#^These authors contributed equally.

*Corresponding authors:

Yun-Lian Lin, Department of Chinese Pharmaceutical Sciences and Chinese Medicine Resources, China Medical University, Taichung 40402, Taiwan. Address: No.91, Hsueh-Shih Road, Taichung 40402, Taiwan. <Tel:+886-4-22053366> ext. 5520; Fax: +886-[4-22078083](mailto:4-22078083); E-mail: [yllin5212@gmail.com](mailto:yllin5212@gmail.com)

Chih-Cheng Chen, Institute of Biomedical Sciences, Academia Sinica, Taipei 115, Taiwan. Address: No.128, Section 2, Academia Road, Taipei 115, Taiwan. <Tel:+886-2-26523917>; Fax: +886-[2-27829224](mailto:2-27829224); E-mail: [chih@ibms.sinica.edu.tw](mailto:chih@ibms.sinica.edu.tw)


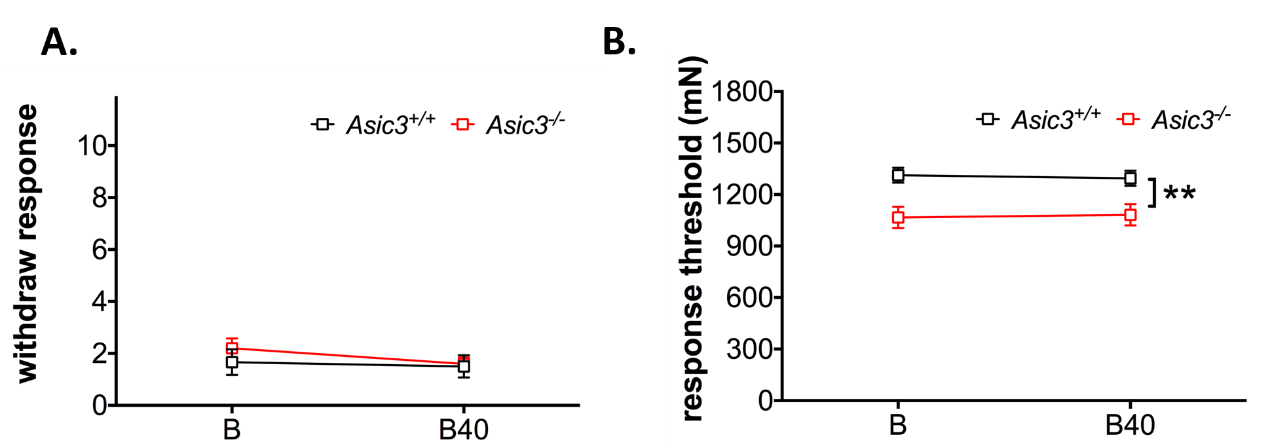


Supplementary Fig 1. Mechanical sensitivity of hind paw and muscle in naïve mice.

(A) Mechanical responses to von Frey filaments and (B) muscle withdrawal thresholds in naïve *Asic3*^+/+^ (n=5) and Asic3^-/-^ mice (n=5). B, baseline mechanical sensitivity measured at 7 to 8 weeks old; B40, baseline mechanical sensitivity measured at 12 to 13 weeks old. **, P < 0.01 between genotypes.


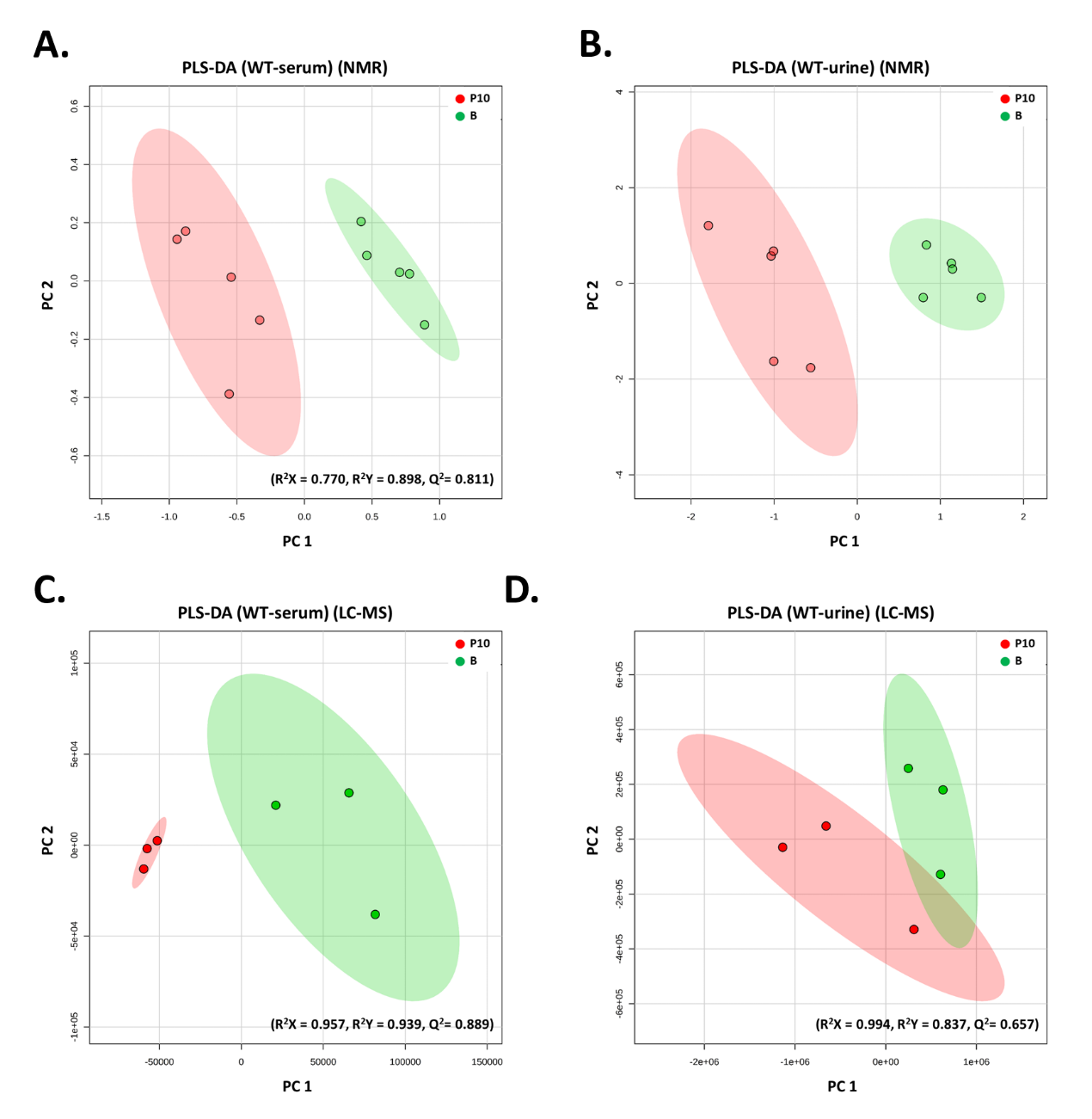


Supplementary Fig 2. PLS-DA analysis for *Asic3^+/+^* mice [ICS-induced experimental fibromyalgia model (P10) vs controls (B)].

^1^H-NMR– and LC-MS/MS–based metabolites were identified by metabolomic comparison between P10 and B. Partial least-squares discriminant analysis (PLS-DA) plots were based on ^1^H-NMR data for serum (A) and urine (B) from B (green) and P10 groups (red) and LC-MS/MS data for serum (C) and urine (D).


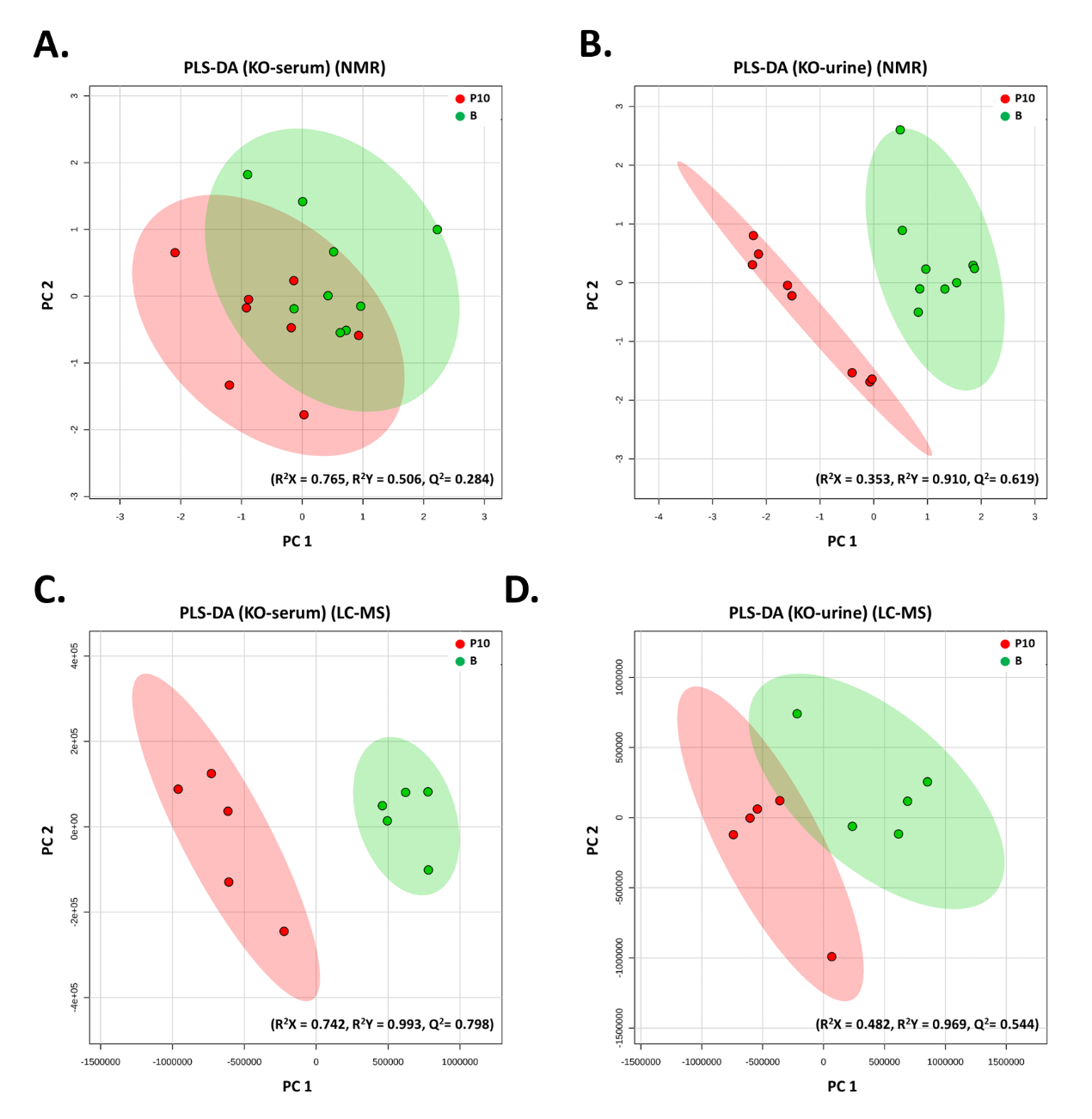


Supplementary Fig 3. PLS-DA analysis for *Asic3^-/-^* mice [ICS-induced experimental fibromyalgia model (P10) vs controls (B)].

^1^H-NMR– and LC-MS/MS–based metabolites were identified by metabolomic comparison between P10 and B. PLS-DA plots were based on ^1^H-NMR data for serum (A) and urine (B) from B (green) and P10 groups (red) and LC-MS/MS data for serum (C) and urine (D).


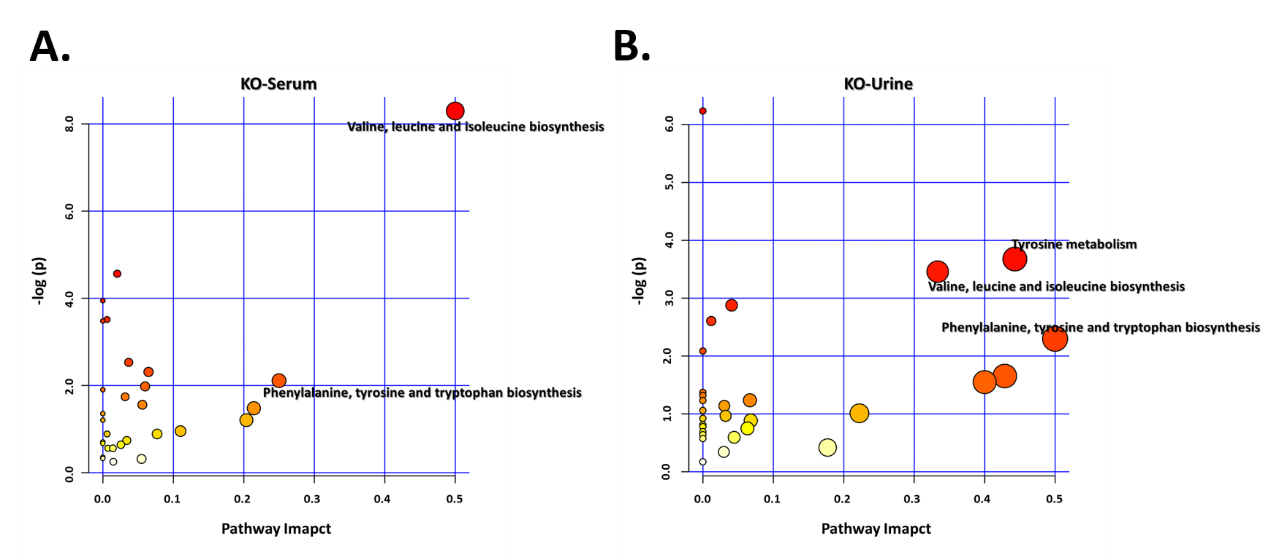


Supplementary Fig 4. Network metabolic pathways associated with ICS-changed metabolites in *Asic3*^-/-^ mice identified by MetPA software.

The metabolism was inferred from changes in levels of intermediates during substance metabolism in serum (A) and urine (B).

Supplementary Table 1. Significant Change in ICS-induced metabolites of WT Serum

|  | Metabolites | PubChem CID | HMDB ID | P10/Basal | | P40/ Basal | |
| --- | --- | --- | --- | --- | --- | --- | --- |
|  |  |  |  | FC | *p* Value | FC | *p* Value (vs P10) |
| LC-MS | Hippuric acid | 464 | HMDB0000714 | 3.58 ± 0.99 | 0.006 | 1.95 ± 0.54 | 0.033 |
|  | Trimethylamine N-oxide | 1145 | HMDB0000925 | 3.33 ± 0.72 | 0.004 | 0.90 ± 0.38 | 0.003 |
|  | Glycerol 3-phosphate ^a^ | 439162 | HMDB0000126 | 2.37 ± 0.41 | 0.021 | 1.71 ± 0.22 | 0.047 |
|  | Hydroxyphenyllactic acid b | 9378 | HMDB0000755 | 2.35 ± 0.35 | 0.031 | 1.66 ± 0.31 | 0.032 |
|  | Succinate ^b^ | 1110 | HMDB0000254 | 1.90 ± 0.02 | 0.039 | 1.39 ± 0.23 | 0.028 |
|  | Butyrylcarnitine ^b^ | 439829 | HMDB0002013 | 1.74 ± 0.19 | 0.047 | 1.61 ± 0.52 | 0.347 |
|  | Isobutyrylcarnitine ^b^ | 10177002 | HMDB0000736 | 1.74 ± 0.19 | 0.047 | 1.61 ± 0.52 | 0.347 |
|  | Catechol | 289 | HMDB0000957 | 1.57 ± 0.05 | 0.045 | 1.02 ± 0.26 | 0.011 |
|  | 4-Methoxyphenylacetic acid ^a, b^ | 7690 | HMDB0002072 | 1.54 ± 0.31 | 0.026 | 0.92 ± 0.08 | 0.041 |
|  | L-3-Phenyllactic acid ^b^ | 643327 | HMDB0000748 | 1.54 ± 0.31 | 0.026 | 0.92 ± 0.08 | 0.041 |
|  | Adenosine | 60961 | HMDB0000050 | 1.49 ± 0.21 | 0.019 | 1.16 ± 0.16 | 0.044 |
|  | Methylmalonate ^a, b^ | 487 | HMDB0000202 | 1.32 ± 0.02 | 0.039 | 1.03 ± 0.23 | 0.028 |
|  | Uridine ^a^ | 6029 | HMDB0000296 | 1.25 ± 0.13 | 0.023 | 0.64 ± 0.32 | 0.049 |
|  | LysoPC (18:3) ^b^ | 24779469 | HMDB0010388 | 1.23 ± 0.06 | 0.033 | 0.98 ± 0.03 | 0.002 |
|  | LysoPC (16:0) | 460602 | HMDB0010382 | 1.19 ± 0.06 | 0.024 | 1.00 ± 0.04 | 0.031 |
|  | LysoPC (14:0) | 460604 | HMDB0010379 | 1.15 ± 0.06 | 0.020 | 0.87 ± 0.11 | 0.010 |
|  | Glucose ^c^ | 5793 | HMDB0000122 | 0.77 ± 0.14 | 0.037 | 1.10 ± 0.10 | 0.013 |
|  | 3-Hydroxybutyric acid | 441 | HMDB0000357 | 0.75 ± 0.08 | 0.047 | 1.23 ± 0.14 | 0.027 |
|  | LysoPC (20:3) ^b^ | 53480467 | HMDB0010393 | 0.73 ± 0.15 | 0.008 | 0.90 ± 0.06 | 0.047 |
|  | LysoPC (20:2) ^b^ | 52924053 | HMDB0010392 | 0.67 ± 0.07 | 0.006 | 0.67 ± 0.05 | 0.498 |
|  | Galacturonic acid ^a^ | 84740 | HMDB0002545 | 0.66 ± 0.19 | 0.030 | 1.13 ± 0.23 | 0.025 |
|  | Glucuronate ^a^ | 444791 | HMDB0000127 | 0.66 ± 0.19 | 0.030 | 1.13 ± 0.23 | 0.025 |
|  | Creatine ^a, b^ | 586 | HMDB0000064 | 0.61 ± 0.19 | 0.037 | 0.74 ± 0.16 | 0.168 |
|  | Carnitine ^b^ | 2724480 | HMDB0000062 | 0.61 ± 0.09 | 0.002 | 0.91 ± 0.11 | 0.016 |
|  | Propionyl-L-carnitine | 107738 | HMDB0000824 | 0.57 ± 0.25 | 0.037 | 1.24 ± 0.54 | 0.216 |
|  | 1-Methyladenosine | 27476 | HMDB0003331 | 0.57 ± 0.13 | 0.006 | 0.81 ± 0.14 | 0.046 |
|  | Decenoyl-L-carnitine ^b^ | - | - | 0.56 ± 0.17 | 0.031 | 1.22 ± 0.01 | 0.003 |
|  | L-Norleucine | 21236 | HMDB0001645 | 0.55 ± 0.13 | 0.023 | 0.95 ± 0.34 | 0.036 |
|  | Tetradecanoyl-L-carnitine | 53477791 | HMDB0005066 | 0.51 ± 0.13 | 0.046 | 0.92 ± 0.24 | 0.031 |
|  | Linoleyl-L-carnitine ^b^ | 129858611 | HMDB0006469 | 0.45 ± 0.14 | 0.028 | 0.86 ± 0.28 | 0.042 |
|  | Dodecanoyl-L-carnitine | 168381 | HMDB0002250 | 0.41 ± 0.11 | 0.039 | 0.73 ± 0.19 | 0.033 |
|  | Oleoyl-L-carnitine | 46907933 | HMDB0005065 | 0.26 ± 0.17 | 0.014 | 0.86 ± 0.42 | 0.044 |
|  | Deoxycholic acid | 222528 | HMDB0000626 | 0.16 ± 0.01 | 0.008 | 1.00 ± 0.06 | 0.003 |
| ^1^H-NMR | Trimethylamine N-oxide ^a, c^ | 1145 | HMDB0000925 | 1.42 ± 0.11 | 0.002 | 0.97 ± 0.13 | 0.002 |
|  | Glucose ^c^ | 5793 | HMDB0000122 | 0.87 ± 0.07 | 0.001 | 1.03 ± 0.09 | 0.005 |
|  | Alanine ^b^ | 5950 | HMDB0000161 | 1.20 ± 0.04 | 0.038 | 1.05 ± 0.06 | 0.067 |

FC: fold change

LysoPC: lysophosphatidylcholine

^a^ Intersection of wild-type mice serum & urine

^b^ Intersection of wild-type mice serum & *Asic3*-knockout serum

^c^ Metabolites were detected in both LC-MS & ^1^H-NMR

^d^ Intersection of *Asic3*-knockout mice serum & urine

^e^ Intersection of wild-type mice urine & *Asic3*-knockout urine

Supplementary Table 2. Significant Change in ICS-induced metabolites of WT Urine

|  | Metabolites | PubChem CID | HMDB ID | P10/Basal | | P40/ Basal | |  |
| --- | --- | --- | --- | --- | --- | --- | --- | --- |
|  |  |  |  | FC | *p* Value | FC | *p* Value (vs P10) |  |
| LC-MS | Methionine | 6137 | HMDB0000696 | 7.52 ± 0.91 | 0.017 | 3.30 ± 0.71 | 0.042 |  |
|  | Dulcitol | 11850 | HMDB0000107 | 5.49 ± 0.76 | 0.018 | 1.51 ± 0.87 | 0.03 |  |
|  | D-Sorbitol | 5780 | HMDB0000247 | 5.49 ± 0.76 | 0.018 | 1.51 ± 0.87 | 0.03 |  |
|  | Mannitol | 6251 | HMDB0000765 | 5.49 ± 0.76 | 0.018 | 1.51 ± 0.87 | 0.03 |  |
|  | Pyroglutamic acid | 7405 | HMDB0000267 | 3.02 ± 0.86 | 0.021 | 1.25 ± 0.34 | 0.026 |  |
|  | D-Ribose 5-phosphate ^e^ | 439167 | HMDB0001548 | 2.92 ± 0.28 | 0.039 | 0.63 ± 0.63 | 0.019 |  |
|  | Creatine ^c, a^ | 586 | HMDB0000064 | 2.56 ± 0.31 | 0.003 | 1.52 ± 0.42 | 0.006 |  |
|  | Deoxycytidine | 13711 | HMDB0000014 | 2.35 ± 0.62 | 0.009 | 0.80 ± 0.23 | 0.006 |  |
|  | Thymidine ^e^ | 5789 | HMDB0000273 | 2.32 ± 0.62 | 0.02 | 0.92 ± 0.21 | 0.046 |  |
|  | 3-Pyridylacetic acid e | 108 | HMDB0001538 | 2.29 ± 0.48 | 0.031 | 0.92 ± 0.39 | 0.014 |  |
|  | P-Hydroxybenzaldehyde | 126 | HMDB0011718 | 2.17 ± 0.23 | 0.005 | 1.20 ± 0.38 | 0.021 |  |
|  | Hydroxykynurenine | 89 | HMDB0000732 | 2.11 ± 0.21 | 0.041 | 0.76 ± 0.36 | 0.007 |  |
|  | Lactic acid ^e^ | 107689 | HMDB0000190 | 2.06 ± 0.21 | 0.045 | 1.03 ± 0.04 | 0.012 |  |
|  | Trimethylamine N-oxide ^a^ | 1145 | HMDB0000925 | 1.94 ± 0.64 | 0.022 | 0.80 ± 0.06 | 0.026 | |
|  | 3'-Sialyllactose P7-20 | - | - | 1.86 ± 0.01 | 0.004 | 1.22 ± 0.11 | 0.002 | |
|  | Glutaric acid ^e^ | 743 | HMDB0000661 | 1.75 ± 0.04 | 0.048 | 0.53 ± 0.31 | 0.011 | |
|  | 4-Methoxyphenylacetic acid ^a^ | 7690 | HMDB0002072 | 1.70 ± 0.59 | 0.001 | 1.25 ± 0.36 | 0.036 | |
|  | Galacturonic acid ^a^ | 84740 | HMDB0002545 | 1.40 ± 0.23 | 0.041 | 0.99 ± 0.12 | 0.029 | |
|  | Glucuronate ^a, e^ | 444791 | HMDB0000127 | 1.40 ± 0.23 | 0.041 | 0.99 ± 0.12 | 0.029 | |
|  | Citric acid ^c, e^ | 311 | HMDB0000094 | 1.34 ± 0.22 | 0.029 | 0.86 ± 0.11 | 0.014 | |
|  | Dopamine ^e^ | 681 | HMDB0000073 | 1.34 ± 0.15 | 0.031 | 1.01 ± 0.15 | 0.028 | |
|  | 5-Methylcytidine ^e^ | 92918 | HMDB0000982 | 1.26 ± 0.19 | 0.047 | 0.98 ± 0.14 | 0.049 | |
|  | 2-Hydroxybutyric acid | 11266 | HMDB0000008 | 1.23 ± 0.01 | 0.031 | 1.08 ± 0.34 | 0.014 | |
|  | 4-Chlorophenylacetate | 13410 | METPA0358 | 1.22 ± 0.09 | 0.019 | 1.00 ± 0.14 | 0.044 | |
|  | D-Fructose 6-phosphate | 69507 | HMDB0000124 | 1.20 ± 0.07 | 0.044 | 0.95 ± 0.12 | 0.057 | |
|  | Glucose 6-phosphate | 5958 | HMDB0001401 | 1.20 ± 0.07 | 0.044 | 0.95 ± 0.12 | 0.057 | |
|  | Uridine ^a^ | 6029 | HMDB0000296 | 0.86 ± 0.03 | 0.046 | 0.70 ± 0.44 | 0.329 | |
|  | 3-Hydroxyisovaleric acid | 69362 | HMDB0000754 | 0.81 ± 0.03 | 0.023 | 1.02 ± 0.08 | 0.007 | |
|  | N-Acetyl-L-tyrosine | 68310 | HMDB0000866 | 0.74 ± 0.22 | 0.02 | 1.22 ± 0.08 | 0.03 | |
|  | 5'-Methylthioadenosine | 439176 | HMDB0001173 | 0.71 ± 0.14 | 0.039 | 1.23 ± 0.30 | 0.027 | |
|  | Phenylacetylglycine ^c, e^ | 68144 | HMDB0000821 | 0.65 ± 0.20 | 0.024 | 1.28 ± 0.21 | 0.009 | |
|  | 4-Aminohippuric acid | 2148 | HMDB0001867 | 0.64 ± 0.01 | 0.042 | 1.11 ± 0.22 | 0.034 | |
|  | 2-Hydroxy-2-methylbutyric acid | 95433 | HMDB0001987 | 0.61 ± 0.08 | 0.025 | 0.73 ± 0.02 | 0.34 | |
|  | Glycerol 3-phosphate ^a^ | 439162 | HMDB0000126 | 0.61 ± 0.02 | 0.023 | 0.88 ± 0.18 | 0.039 | |
|  | Histamine | 774 | HMDB0000870 | 0.55 ± 0.24 | 0.049 | 0.96 ± 0.17 | 0.036 | |
|  | *cis*-Aconitate | 643757 | HMDB0000072 | 0.53 ± 0.01 | 0.003 | 0.84 ± 0.11 | 0.002 | |
|  | 3-Methoxytyramine | 1669 | HMDB0000022 | 0.52 ± 0.21 | 0.048 | 1.08 ± 0.32 | 0.031 | |
|  | Spermine | 1103 | HMDB0001256 | 0.41 ± 0.18 | 0.026 | 1.09 ± 0.46 | 0.039 | |
|  | 3-Hydroxytetradecanolycarnitine | - | - | 0.40 ± 0.19 | 0.028 | 0.57 ± 0.07 | 0.119 | |
|  | N6-Methyladenine | 67955 | HMDB0002099 | 0.40 ± 0.01 | 0.038 | 1.16 ± 0.36 | 0.05 | |
|  | 3-Hexenedioic acid | 5351896 | HMDB0000393 | 0.35 ± 0.11 | 0.052 | 0.31± 0.10 | 0.289 | |
|  | Kynurenate | 3845 | HMDB0000715 | 0.32 ± 0.19 | 0.045 | 0.69 ± 0.19 | 0.044 | |
|  | Sebacic acid | 5192 | HMDB0000792 | 0.27 ± 0.11 | 0.04 | 0.42 ± 0.07 | 0.049 | |
|  | Tiglyl-L-carnitine | 91825636 | HMDB0002366 | 0.26 ± 0.01 | 0.011 | 2.34 ± 0.57 | 0.029 | |
| ^1^H-NMR | Taurine ^e^ | 1123 | HMDB0000251 | 1.58 ± 0.37 | 0.019 | 0.96 ± 0.18 | 0.010 | |
|  | Citric acid ^c, e^ | 311 | HMDB0000094 | 1.42 ± 0.19 | 0.018 | 0.74 ± 0.08 | 0.007 | |
|  | Creatine ^c, a^ | 586 | HMDB0000064 | 1.43 ± 0.13 | 0.007 | 1.11 ± 0.09 | 0.046 | |
|  | Phenylacetylglycine ^c, e^ | 68144 | HMDB0000821 | 0.77 ± 0.06 | 0.001 | 1.04 ± 0.11 | 0.001 | |
|  | Methylmalonate ^a, e^ | 487 | HMDB0000202 | 0.66 ± 0.04 | 0.001 | 0.98 ± 0.05 | 0.001 | |
|  | Trigonelline | 5570 | HMDB0000875 | 0.58 ± 0.01 | 0.001 | 0.87 ± 0.02 | 0.001 | |

FC: fold change

^a^ Intersection of wild-type mice serum & urine

^b^ Intersection of wild-type mice serum & *Asic3*-knockout serum

^c^ Metabolites were detected in both LC-MS & ^1^H-NMR

^d^ Intersection of *Asic3*-knockout mice serum & urine

^e^ Intersection of wild-type mice urine & *Asic3*-knockout urine

Supplementary Table 3. Significant Change in ICS-induced metabolites of KO Serum

|  | Metabolites | PubChem CID | HMDB ID | P10/Basal | | P40/ Basal | |
| --- | --- | --- | --- | --- | --- | --- | --- |
|  |  |  |  | FC | *p* Value | FC | *p* Value (vs P10) |
| LC-MS | 5-Methylcytidine ^d^ | 92918 | HMDB0000982 | 3.04 ± 0.68 | 0.001 | 2.37 ± 0.46 | 0.053 |
|  | Pyroglutamic acid | 7405 | HMDB0000267 | 2.99 ± 1.03 | 0.002 | 1.85 ± 0.47 | 0.027 |
|  | 2-Methylbutyryl-L-carnitine | 9881339 | HMDB0000378 | 2.83 ± 0.93 | 0.003 | 1.39 ± 0.38 | 0.007 |
|  | Propionyl-L-carnitine ^b^ | 107738 | HMDB0000824 | 2.57 ± 0.98 | 0.014 | 2.11 ± 0.76 | 0.435 |
|  | Phenylethanolamine | 1000 | HMDB0001065 | 2.43 ± 0.90 | 0.004 | 2.80 ± 0.84 | 0.258 |
|  | 3-Methyladenine | 1673 | HMDB0011600 | 2.39 ± 0.38 | 0.002 | 1.78 ± 0.41 | 0.048 |
|  | 1-Methyladenine | 78821 | HMDB0011599 | 2.39 ± 0.38 | 0.002 | 1.78 ± 0.41 | 0.048 |
|  | Hexanoylcarnitine ^d^ | 6426853 | HMDB0000705 | 2.38 ± 0.35 | 0.001 | 1.60 ± 0.47 | 0.009 |
|  | N-Acetyl-L-phenylalanine | 74839 | HMDB0000512 | 2.29 ± 0.14 | 0.002 | 1.74 ± 0.31 | 0.053 |
|  | Carnitine ^b, d^ | 2724480 | HMDB0000062 | 2.25 ± 0.52 | 0.002 | 1.89 ± 0.49 | 0.304 |
|  | Isovaleryl-L-carnitine | 169235 | HMDB0000688 | 2.24 ± 0.25 | 0.001 | 1.51 ± 0.35 | 0.003 |
|  | Valeryl-L-carnitine | 53481619 | HMDB0013128 | 2.24 ± 0.25 | 0.001 | 1.51 ± 0.35 | 0.003 |
|  | Butyrylcarnitine ^b, d^ | 439829 | HMDB0002013 | 2.23 ± 0.71 | 0.005 | 1.75 ± 0.39 | 0.111 |
|  | Isobutyrylcarnitine ^b, d^ | 10177002 | HMDB0000736 | 2.23 ± 0.71 | 0.005 | 1.75 ± 0.39 | 0.111 |
|  | N-Methylhydantoin | 69217 | HMDB0003646 | 2.18 ± 0.43 | 0.002 | 1.69 ± 0.43 | 0.051 |
|  | 1-Phenylethylamine | 7408 | HMDB0002017 | 2.16 ± 0.58 | 0.002 | 1.91 ± 0.33 | 0.218 |
|  | Methionine ^c^ | 6137 | HMDB0000696 | 2.15 ± 0.32 | 0.001 | 2.02 ± 0.78 | 0.376 |
|  | 2-Hydroxycaproic acid | 99824 | HMDB0001624 | 2.14 ± 0.56 | 0.004 | 1.51 ± 0.48 | 0.050 |
|  | Taurine ^d^ | 1123 | HMDB0000251 | 2.13 ± 0.26 | 0.006 | 1.86 ± 0.27 | 0.176 |
|  | Octanoyl-L-carnitine | 11953814 | HMDB0000791 | 2.13 ± 0.54 | 0.012 | 1.35 ± 0.57 | 0.037 |
|  | Eicosenoyl-L-carnitine | - | - | 2.13 ± 0.45 | 0.040 | 1.25 ± 0.09 | 0.056 |
|  | Acetylcarnitine | 1 | HMDB0000201 | 2.10 ± 0.42 | 0.001 | 1.82 ± 0.49 | 0.180 |
|  | Dihydrouracil | 649 | HMDB0000076 | 2.09 ± 0.15 | 0.001 | 1.67 ± 0.39 | 0.028 |
|  | Linoleyl-L-carnitine ^b^ | 129858611 | HMDB0006469 | 2.05 ± 0.42 | 0.005 | 1.34 ± 0.16 | 0.004 |
|  | Stearoyl-L-carnitine | 6426855 | HMDB0000848 | 2.05 ± 0.63 | 0.010 | 1.39 ± 0.37 | 0.047 |
|  | Phenylalanine | 6140 | HMDB0000159 | 2.04 ± 0.51 | 0.002 | 2.08 ± 0.09 | 0.431 |
|  | 3-Hydroxymethylglutaric acid | 1662 | HMDB0000355 | 2.03 ± 0.85 | 0.035 | 1.19 ± 0.01 | 0.150 |
|  | L-3-Phenyllactic acid ^b^ | 444718 | HMDB0000748 | 1.98 ± 0.32 | 0.005 | 1.58 ± 0.12 | 0.024 |
|  | 4-Methoxyphenylacetic acid ^b^ | 7690 | HMDB0002072 | 1.98 ± 0.32 | 0.005 | 1.58 ± 0.12 | 0.024 |
|  | Desaminotyrosine | 10394 | HMDB0002199 | 1.98 ± 0.32 | 0.005 | 1.58 ± 0.12 | 0.024 |
|  | L-Kynurenine | 161166 | HMDB0000684 | 1.98 ± 0.49 | 0.001 | 1.69 ± 0.33 | 0.147 |
|  | Thymidine ^d^ | 5789 | HMDB0000273 | 1.97 ± 0.43 | 0.002 | 1.53 ± 0.27 | 0.044 |
|  | 4-Guanidinobutanoic acid | 500 | HMDB0003464 | 1.97 ± 0.30 | 0.001 | 1.51 ± 0.24 | 0.016 |
|  | Selenomethionine | 15103 | HMDB0003966 | 1.96 ± 0.18 | 0.002 | 1.18 ± 0.28 | 0.040 |
|  | Creatine ^b^ | 586 | HMDB0000064 | 1.95 ± 0.21 | 0.000 | 1.68 ± 0.25 | 0.054 |
|  | Decenoyl-L-carnitine ^b, d^ | - | - | 1.95 ± 0.30 | 0.003 | 1.46 ± 0.42 | 0.040 |
|  | Tetradecenoyl-L-carnitine | 53481677 | HMDB0013329 | 1.94 ± 0.43 | 0.014 | 1.45 ± 0.24 | 0.030 |
|  | 5'-Methylthioadenosine | 439176 | HMDB0001173 | 1.93 ± 0.27 | 0.001 | 1.63 ± 0.13 | 0.033 |
|  | N-Acetyl-L-alanine | 88064 | HMDB0000766 | 1.82 ± 0.13 | 0.001 | 1.45 ± 0.22 | 0.048 |
|  | Betaine ^d^ | 247 | HMDB0000043 | 1.82 ± 0.19 | 0.000 | 1.59 ± 0.29 | 0.072 |
|  | Valine ^c, d^ | 6287 | HMDB0000883 | 1.82 ± 0.19 | 0.000 | 1.59 ± 0.29 | 0.072 |
|  | Uracil | 1174 | HMDB0000300 | 1.82 ± 0.22 | 0.001 | 1.57 ± 0.14 | 0.035 |
|  | Oleanolic acid | 10494 | HMDB0002364 | 1.81 ± 0.45 | 0.034 | 0.79 ± 0.32 | 0.015 |
|  | Histamine | 774 | HMDB0000870 | 1.74 ± 0.29 | 0.011 | 1.62 ± 0.26 | 0.296 |
|  | 3-Indolepropionic acid | 3744 | HMDB0002302 | 1.68 ± 0.43 | 0.051 | 2.11 ± 0.74 | 0.168 |
|  | Isoleucine ^c^ | 6306 | HMDB0000172 | 1.64 ± 0.17 | 0.001 | 1.56 ± 0.10 | 0.201 |
|  | Hexadecanoyl-L-carnitine | 11953816 | HMDB0000222 | 1.64 ± 0.49 | 0.024 | 1.23 ± 0.42 | 0.099 |
|  | 2-Oxoglutarate ^d^ | 51 | HMDB0000208 | 1.63 ± 0.39 | 0.010 | 1.28 ± 0.06 | 0.042 |
|  | Decanoylcarnitine | 10245190 | HMDB0000651 | 1.61 ± 0.26 | 0.013 | 1.25 ± 0.18 | 0.050 |
|  | Pyrrolidonecarboxylic acid | 499 | HMDB0000805 | 1.60 ± 0.44 | 0.033 | 1.17 ± 0.21 | 0.041 |
|  | Rhamnose ^d^ | 25310 | HMDB0000849 | 1.56 ± 0.31 | 0.007 | 1.87 ± 0.81 | 0.251 |
|  | g-Glutamylleucine | 151023 | HMDB0011171 | 1.53 ± 0.18 | 0.016 | 1.54 ± 0.23 | 0.461 |
|  | D-Glucurono-6,3-lactone | 2724333 | HMDB0006355 | 1.50 ± 0.35 | 0.017 | 1.01 ± 0.23 | 0.016 |
|  | Dopamine ^d^ | 681 | HMDB0000073 | 1.49 ± 0.35 | 0.020 | 1.18 ± 0.20 | 0.063 |
|  | Hydroxyphenyllactic acid ^b, d^ | 9378 | HMDB0000755 | 1.46 ± 0.27 | 0.012 | 1.43 ± 0.29 | 0.455 |
|  | Hydroxyoctanoic acid | 94180 | HMDB0000711 | 1.42 ± 0.16 | 0.018 | 1.05 ± 0.29 | 0.032 |
|  | LysoPC (16:1) | 24779461 | HMDB0010383 | 1.39 ± 0.21 | 0.019 | 0.99 ± 0.18 | 0.024 |
|  | LysoPC (18:3) ^b^ | 24779469 | HMDB0010388 | 1.38 ± 0.17 | 0.015 | 1.01 ± 0.20 | 0.019 |
|  | LysoPC (20:3) ^b^ | 53480467 | HMDB0010393 | 1.38 ± 0.23 | 0.010 | 0.93 ± 0.15 | 0.003 |
|  | Methylmalonate ^b, c, d^ | 487 | HMDB0000202 | 1.36 ± 0.14 | 0.042 | 1.05 ± 0.17 | 0.010 |
|  | Ascorbic Acid | 54670067 | HMDB0000044 | 1.32 ± 0.19 | 0.034 | 0.90 ± 0.21 | 0.006 |
|  | Ketoleucine | 70 | HMDB0000695 | 1.31 ± 0.19 | 0.017 | 1.14 ± 0.17 | 0.091 |
|  | 2-Ketohexanoic acid | 159664 | HMDB0001864 | 1.31 ± 0.19 | 0.017 | 1.14 ± 0.17 | 0.091 |
|  | LysoPC (20:2) ^b^ | 52924053 | HMDB0010392 | 1.29 ± 0.22 | 0.028 | 0.85 ± 0.31 | 0.016 |
|  | LysoPC (20:1) | 52924051 | HMDB0010391 | 1.21 ± 0.10 | 0.031 | 0.84 ± 0.15 | 0.002 |
|  | Succinate ^b, c^ | 1110 | HMDB0000254 | 1.20 ± 0.17 | 0.031 | 0.88 ± 0.29 | 0.038 |
|  | 4-Coumarate | 637542 | HMDB0002035 | 0.70 ± 0.12 | 0.022 | 1.05 ± 0.11 | 0.007 |
| ^1^H-NMR | Succinate ^b, c^ | 1110 | HMDB0000254 | 2.03 ± 0.16 | 0.005 | 1.72 ± 0.06 | 0.213 |
|  | Methionine ^c^ | 6137 | HMDB0000696 | 1.51 ± 0.07 | 0.002 | 1.45 ± 0.15 | 0.397 |
|  | Isoleucine ^c^ | 6306 | HMDB0000172 | 1.46 ± 0.14 | 0.027 | 0.97 ± 0.07 | 0.026 |
|  | Methylmalonate ^b, c, d^ | 487 | HMDB0000202 | 1.44 ± 0.09 | 0.029 | 1.08 ± 0.15 | 0.052 |
|  | Valine ^c, d^ | 6287 | HMDB0000883 | 1.42 ± 0.06 | 0.001 | 1.18 ± 0.07 | 0.043 |
|  | Acetate | 176 | HMDB0000042 | 1.37 ± 0.13 | 0.028 | 0.97 ± 0.07 | 0.032 |
|  | Leucine | 6106 | HMDB0000687 | 1.22 ± 0.07 | 0.042 | 1.15 ± 0.07 | 0.277 |
|  | Alanine ^b^ | 5950 | HMDB0000161 | 0.86 ± 0.06 | 0.029 | 1.02 ± 0.07 | 0.021 |

FC: fold change

LysoPC: lysophosphatidylcholine

^a^ Intersection of wild-type mice serum & urine

^b^ Intersection of wild-type mice serum & *Asic3*-knockout serum

^c^ Metabolites were detected in both LC-MS & ^1^H-NMR

^d^ Intersection of *Asic3*-knockout mice serum & urine

^e^ Intersection of wild-type mice urine & *Asic3*-knockout urine

Supplementary Table 4. Significant Change in ICS-induced metabolites of KO Urine

|  | Metabolites | PubChem CID | HMDB ID | P10/Basal | | P40/ Basal | |
| --- | --- | --- | --- | --- | --- | --- | --- |
|  |  |  |  | FC | *p* Value | FC | *p* Value (vs P10) |
| LC-MS | Malic Acid | 525 | HMDB0000744 | 4.59 ± 1.76 | 0.005 | 0.92 ± 0.12 | 0.004 |
|  | Lactic acid ^e^ | 107689 | HMDB0000190 | 4.51 ± 0.21 | 0.04 | 2.93 ± 0.16 | 0.042 |
|  | Citric acid ^c, e^ | 311 | HMDB0000094 | 3.40 ± 0.63 | 0.008 | 1.01 ± 0.11 | 0.001 |
|  | Betaine ^d^ | 247 | HMDB0000043 | 3.13 ± 0.45 | 0.012 | 1.67 ± 0.30 | 0.008 |
|  | Valine ^d^ | 6287 | HMDB0000883 | 3.13 ± 0.45 | 0.012 | 1.67 ± 0.30 | 0.008 |
|  | Nicotinate | 938 | HMDB0001488 | 3.03 ± 0.99 | 0.005 | 1.76 ± 0.64 | 0.039 |
|  | Glutaric acid ^e^ | 743 | HMDB0000661 | 2.95 ± 0.57 | 0.004 | 1.26 ± 0.14 | 0.001 |
|  | Glycolic acid | 757 | HMDB0000115 | 2.83 ± 0.09 | 0.003 | 0.61 ± 0.01 | 0.001 |
|  | Picolinic acid | 1018 | HMDB0002243 | 2.63 ± 1.32 | 0.022 | 1.29 ± 0.47 | 0.042 |
|  | 2-Oxoglutarate ^d^ | 51 | HMDB0000208 | 2.28 ± 0.55 | 0.023 | 0.84 ± 0.39 | 0.041 |
|  | Itaconic acid | 811 | HMDB0002092 | 2.22 ± 0.68 | 0.04 | 1.03 ± 0.15 | 0.028 |
|  | Methylmalonate ^d, e^ | 487 | HMDB0000202 | 2.21 ± 0.46 | 0.044 | 1.13 ± 0.61 | 0.051 |
|  | 3-Pyridylacetic acid ^e^ | 108 | HMDB0001538 | 1.99 ± 0.52 | 0.014 | 1.16 ± 0.60 | 0.035 |
|  | L-Citrulline | 9750 | HMDB0000904 | 1.84 ± 0.23 | 0.004 | 1.22 ± 0.52 | 0.042 |
|  | Hydroxyphenyllactic acid ^d^ | 9378 | HMDB0000755 | 1.66 ± 0.61 | 0.027 | 1.04 ± 0.18 | 0.029 |
|  | D-Ribose 5-phosphate ^e^ | 439167 | HMDB0001548 | 1.60 ± 0.39 | 0.012 | 0.87 ± 0.10 | 0.002 |
|  | Phosphorylcholine | 1014 | HMDB0001565 | 1.54 ± 0.55 | 0.033 | 1.02 ± 0.13 | 0.056 |
|  | 4-Hydroxybenzoate | 135 | HMDB0000500 | 1.52 ± 0.27 | 0.004 | 0.96 ± 0.21 | 0.003 |
|  | Tyrosine | 6057 | HMDB0000158 | 1.47 ± 0.39 | 0.042 | 1.01 ± 0.30 | 0.014 |
|  | Glycylproline | 79101 | HMDB0000721 | 1.45 ± 0.34 | 0.012 | 1.41 ± 0.29 | 0.429 |
|  | 1,3,7-Trimethyluric acid | 79437 | HMDB0002123 | 1.32 ± 0.10 | 0.019 | 0.81 ± 0.18 | 0.001 |
|  | 5-Methylcytidine ^d, e^ | 92918 | HMDB0000982 | 1.31 ± 0.15 | 0.016 | 1.64 ± 0.22 | 0.018 |
|  | Diethylthiophosphate | 655 | HMDB0001460 | 1.27 ± 0.25 | 0.04 | 0.65 ± 0.30 | 0.005 |
|  | L-Tryptophan | 6305 | HMDB0000929 | 1.16 ± 0.08 | 0.034 | 1.04 ± 0.15 | 0.039 |
|  | 3-Hydroxymandelic acid | 86957 | HMDB0000750 | 0.80 ± 0.15 | 0.047 | 1.08 ± 0.12 | 0.011 |
|  | Homogentistic acid | 780 | HMDB0000130 | 0.80 ± 0.15 | 0.047 | 1.08 ± 0.12 | 0.011 |
|  | Levulinic acid | 11579 | HMDB0000720 | 0.79 ± 0.21 | 0.047 | 1.50 ± 0.41 | 0.041 |
|  | p-Aminobenzoic acid | 978 | HMDB0001392 | 0.79 ± 0.20 | 0.036 | 1.00 ± 0.16 | 0.054 |
|  | Phenylacetylglycine ^c, e^ | 68144 | HMDB0000821 | 0.78 ± 0.09 | 0.032 | 1.10 ± 0.24 | 0.014 |
|  | 3-Hydroxydodecanoyl-L-carnitine | - | - | 0.74 ± 0.26 | 0.041 | 0.67 ± 0.19 | 0.326 |
|  | Dopamine ^d, e^ | 681 | HMDB0000073 | 0.74 ± 0.19 | 0.051 | 1.07 ± 0.30 | 0.036 |
|  | Thymidine ^d, e^ | 5789 | HMDB0000273 | 0.71 ± 0.17 | 0.017 | 0.71 ± 0.14 | 0.477 |
|  | Glucuronate ^e^ | 444791 | HMDB0000127 | 0.70 ± 0.36 | 0.049 | 0.58 ± 0.30 | 0.323 |
|  | 2-Isopropylmalic acid | 5280523 | HMDB0000402 | 0.68 ± 0.13 | 0.008 | 1.03 ± 0.10 | 0.044 |
|  | Carnitine ^c, d^ | 2724480 | HMDB0000062 | 0.67 ± 0.26 | 0.045 | 1.11 ± 0.38 | 0.153 |
|  | Rhamnose ^d^ | 25310 | HMDB0000849 | 0.67 ± 0.26 | 0.02 | 0.92 ± 0.11 | 0.048 |
|  | Azelaic acid | 2266 | HMDB0000784 | 0.66 ± 0.01 | 0.033 | 0.83 ± 0.10 | 0.054 |
|  | DL-Dopa | 836 | HMDB0000609 | 0.64 ± 0.20 | 0.048 | 0.94 ± 0.13 | 0.025 |
|  | Taurine ^c, d, e^ | 1123 | HMDB0000251 | 0.63 ± 0.22 | 0.039 | 1.02 ± 0.31 | 0.021 |
|  | Salicin | 439503 | HMDB0003546 | 0.63 ± 0.13 | 0.04 | 0.52 ± 0.01 | 0.001 |
|  | Decenoyl-L-carnitine ^d^ | - | - | 0.46 ± 0.15 | 0.023 | 1.47 ± 0.17 | 0.003 |
|  | Hexanoylcarnitine ^d^ | 6426853 | HMDB0000705 | 0.45 ± 0.12 | 0.016 | 0.99 ± 0.67 | 0.053 |
|  | 2-Furoylglycine | 21863 | HMDB0000439 | 0.41 ± 0.17 | 0.012 | 0.76 ± 0.51 | 0.038 |
|  | Isobutyrylcarnitine ^d^ | 10177002 | HMDB0000736 | 0.40 ± 0.18 | 0.026 | 0.98 ± 0.45 | 0.036 |
|  | Butyrylcarnitine ^d^ | 439829 | HMDB0002013 | 0.39 ± 0.12 | 0.022 | 0.98 ± 0.29 | 0.012 |
|  | Acetylglycine | 10972 | HMDB0000532 | 0.36 ± 0.07 | 0.049 | 0.94 ± 0.16 | 0.014 |
|  | trans-Aconitic acid | 444212 | HMDB0000958 | 0.33 ± 0.13 | 0.037 | 0.89 ± 0.59 | 0.037 |
|  | Salicyluric acid | 10253 | HMDB0000840 | 0.27 ± 0.05 | 0.033 | 0.64 ± 0.64 | 0.072 |
|  | Urea | 1176 | HMDB0000294 | 6.03 ± 0.25 | 0.002 | 1.99 ± 0.59 | 0.039 |
| ^1^H-NMR | Citric acid ^c, e^ | 311 | HMDB0000094 | 1.44 ± 0.16 | 0.045 | 0.74 ± 0.22 | 0.020 |
|  | Phenylacetylglycine ^c, e^ | 68144 | HMDB0000821 | 0.79 ± 0.13 | 0.046 | 1.01 ± 0.15 | 0.095 |
|  | Taurine ^c, d, e^ | 1123 | HMDB0000251 | 0.72 ± 0.10 | 0.015 | 0.91 ± 0.05 | 0.038 |
|  | N-Carbamoyl-b-alanine | 111 | HMDB0000026 | 0.71 ± 0.14 | 0.011 | 0.75 ± 0.08 | 0.394 |
|  | 2-oxoisocaproate | 70 | HMDB0000695 | 0.69 ± 0.11 | 0.006 | 0.82 ± 0.08 | 0.126 |
|  | Trimethylamine-N-oxide | 1145 | HMDB0000925 | 0.66 ± 0.12 | 0.002 | 0.87 ± 0.06 | 0.041 |
|  | Carnitine ^c, d^ | 2724480 | HMDB0000062 | 0.49 ± 0.17 | 0.000 | 0.75 ± 0.12 | 0.036 |

FC: fold change

^a^ Intersection of wild-type mice serum & urine

^b^ Intersection of wild-type mice serum & *Asic3-*knockout serum

^c^ Metabolites were detected in both LC-MS & ^1^H-NMR

^d^ Intersection of *Asic3*-knockout mice serum & urine

^e^ Intersection of wild-type mice urine & *Asic3*-knockout urine
